# Supplementary material for: The African swine fever virus protease pS273R inhibits DNA sensing cGAS-STING pathway by targeting IKKε
Source: Virulence. 2022 May 1;13(1):740–56. doi: 10.1080/21505594.2022.2065962 (PMC9067533; doi:10.1080/21505594.2022.2065962)
Supplement: Supplemental Material [file KVIR_A_2065962_SM4607.zip › supplementary/Raw data of TCID50-PAM cells.pdf]

VSV（MOI 0.01）$TCID_{50}$

Vector

Virus dilution	Number of inoculated cell tubes	Number of cells without lesion	Number of diseased cell tubes	Cumulative total		Proportion of cytopathic ducts	Incidence of cytopathy
				Non diseased tube	diseased tube		
10^{-1}	4	0	4	0	27	27/27	100%
10^{-2}	4	0	4	0	23	23/23	100%
10^{-3}	4	0	4	0	19	19/19	100%
10^{-4}	4	0	4	0	15	15/15	100%
10^{-5}	4	0	4	0	11	11/11	100%
10^{-6}	4	0	4	0	7	7/7	100%
10^{-7}	4	2	2	2	3	3/5	60%
10^{-8}	4	3	1	5	1	1/6	16.7%
10^{-9}	4	4	0	9	0	0/9	0%
10^{-10}	4	4	0	13	0	0/13	0%
Control	4	4	0				

VSV（MOI 0.01）$TCID_{50}$

S273R

Virus dilution	Number of inoculated cell tubes	Number of cells without lesion	Number of diseased cell tubes	Cumulative total		Proportion of cytopathic ducts	Incidence of cytopathy
				Non diseased tube	diseased tube		
10^{-1}	4	0	4	0	37	37/37	100%
10^{-2}	4	0	4	0	33	33/33	100%
10^{-3}	4	0	4	0	29	29/29	100%
10^{-4}	4	0	4	0	25	25/25	100%
10^{-5}	4	0	4	0	21	21/21	100%
10^{-6}	4	0	4	0	17	17/17	100%
10^{-7}	4	0	4	0	13	13/13	100%
10^{-8}	4	0	4	0	9	9/9	100%
10^{-9}	4	1	3	1	5	5/6	83.3%
10^{-10}	4	2	2	3	2	2/5	40%
Control	4	4	0				

VSV（MOI 0.001）$TCID_{50}$

Vector

Virus dilution	Number of inoculated cell tubes	Number of cells without lesion	Number of diseased cell tubes	Cumulative total		Proportion of cytopathic ducts	Incidence of cytopathy
				Non diseased tube	diseased tube		
10^{-1}	4	0	4	0	16	16/16	100%
10^{-2}	4	0	4	0	12	12/12	100%
10^{-3}	4	0	4	0	8	8/8	100%
10^{-4}	4	1	3	1	4	4/5	80%
10^{-5}	4	3	1	4	1	1/5	20%
10^{-6}	4	4	0	8	0	0/8	0%
10^{-7}	4	4	0	12	0	0/12	0%
10^{-8}	4	4	0	16	0	0/16	0%
10^{-9}	4	4	0	20	0	0/20	0%
10^{-10}	4	4	0	24	0	0/24	0%
Control	4	4	0				

VSV（MOI0.001）$TCID_{50}$

S273R

Virus dilution	Number of inoculated cell tubes	Number of cells without lesion	Number of diseased cell tubes	Cumulative total		Proportion of cytopathic ducts	Incidence of cytopathy
				Non diseased tube	diseased tube		
10^{-1}	4	0	4	0	23	23/23	100%
10^{-2}	4	0	4	0	19	19/19	100%
10^{-3}	4	0	4	0	15	15/15	100%
10^{-4}	4	0	4	0	11	11/11	100%
10^{-5}	4	0	4	0	7	7/7	100%
10^{-6}	4	2	2	2	3	3/5	60%
10^{-7}	4	3	1	5	1	1/6	16.7%
10^{-8}	4	4	0	9	0	0/9	0%
10^{-9}	4	4	0	13	0	0/13	0%
10^{-10}	4	4	0	17	0	0/17	0%
Control	4	4	0				

HSV（MOI 0.1）$TCID_{50}$

Vector

Virus dilution	Number of inoculated cell tubes	Number of cells without lesion	Number of diseased cell tubes	Cumulative total		Proportion of cytopathic ducts	Incidence of cytopathy
				Non diseased tube	diseased tube		
10^{-1}	4	0	4	0	19	19/19	100%
10^{-2}	4	0	4	0	15	15/15	100%
10^{-3}	4	0	4	0	11	11/11	100%
10^{-4}	4	1	3	1	7	7/8	87.5%
10^{-5}	4	1	3	2	4	4/6	66.7%
10^{-6}	4	3	1	5	1	1/6	16.7%
10^{-7}	4	4	0	9	0	0/9	0%
10^{-8}	4	4	0	13	0	0/13	0%
10^{-9}	4	4	0	17	0	0/17	0%
10^{-10}	4	4	0	21	0	0/21	0%
Control	4	4	0				

HSV（MOI 0.1）$TCID_{50}$

S273R

Virus dilution	Number of inoculated cell tubes	Number of cells without lesion	Number of diseased cell tubes	Cumulative total		Proportion of cytopathic ducts	Incidence of cytopathy
				Non diseased tube	diseased tube		
10^{-1}	4	0	4	0	29	29/29	100%
10^{-2}	4	0	4	0	25	25/25	100%
10^{-3}	4	0	4	0	21	21/21	100%
10^{-4}	4	0	4	0	17	17/17	100%
10^{-5}	4	0	4	0	13	13/13	100%
10^{-6}	4	0	4	0	9	9/9	100%
10^{-7}	4	1	3	1	5	5/6	83.3%
10^{-8}	4	2	2	3	2	2/5	40%
10^{-9}	4	4	0	7	0	0/7	0%
10^{-10}	4	4	0	11	0	0/11	0%
Control	4	4	0				

HSV（MOI 0.01）$TCID_{50}$

Vector

Virus dilution	Number of inoculated cell tubes	Number of cells without lesion	Number of diseased cell tubes	Cumulative total		Proportion of cytopathic ducts	Incidence of cytopathy
				Non diseased tube	diseased tube		
10^{-1}	4	0	4	0	15	15/15	100%
10^{-2}	4	0	4	0	11	11/11	100%
10^{-3}	4	0	4	0	7	7/7	100%
10^{-4}	4	2	2	2	3	3/5	60%
10^{-5}	4	3	1	5	1	1/6	16,7%
10^{-6}	4	4	0	9	0	0/9	0%
10^{-7}	4	4	0	13	0	0/13	0%
10^{-8}	4	4	0	17	0	0/17	0%
10^{-9}	4	4	0	21	0	0/21	0%
10^{-10}	4	4	0	25	0	0/25	0%
Control	4	4	0				

HSV（MOI0.01）$TCID_{50}$

S273R

Virus dilution	Number of inoculated cell tubes	Number of cells without lesion	Number of diseased cell tubes	Cumulative total		Proportion of cytopathic ducts	Incidence of cytopathy
				Non diseased tube	diseased tube		
10^{-1}	4	0	4	0	23	23/23	100%
10^{-2}	4	0	4	0	19	19/19	100%
10^{-3}	4	0	4	0	15	15/15	100%
10^{-4}	4	0	4	0	11	11/11	100%
10^{-5}	4	1	3	1	7	7/8	87.5%
10^{-6}	4	1	3	2	4	4/6	66.7%
10^{-7}	4	3	1	5	1	1/6	16.7%
10^{-8}	4	4	0	9	0	0/9	0%
10^{-9}	4	4	0	13	0	0/13	0%
10^{-10}	4	4	0	17	0	0/17	0%
Control	4	4	0				
