# Supplementary material for: The African swine fever virus protease pS273R inhibits DNA sensing cGAS-STING pathway by targeting IKKε
Source: Virulence. 2022 May 1;13(1):740–56. doi: 10.1080/21505594.2022.2065962 (PMC9067533; doi:10.1080/21505594.2022.2065962)
Supplement: Supplemental Material [file KVIR_A_2065962_SM4607.zip › supplementary/Raw data of WB and microscopy images.pdf]

FIG1C

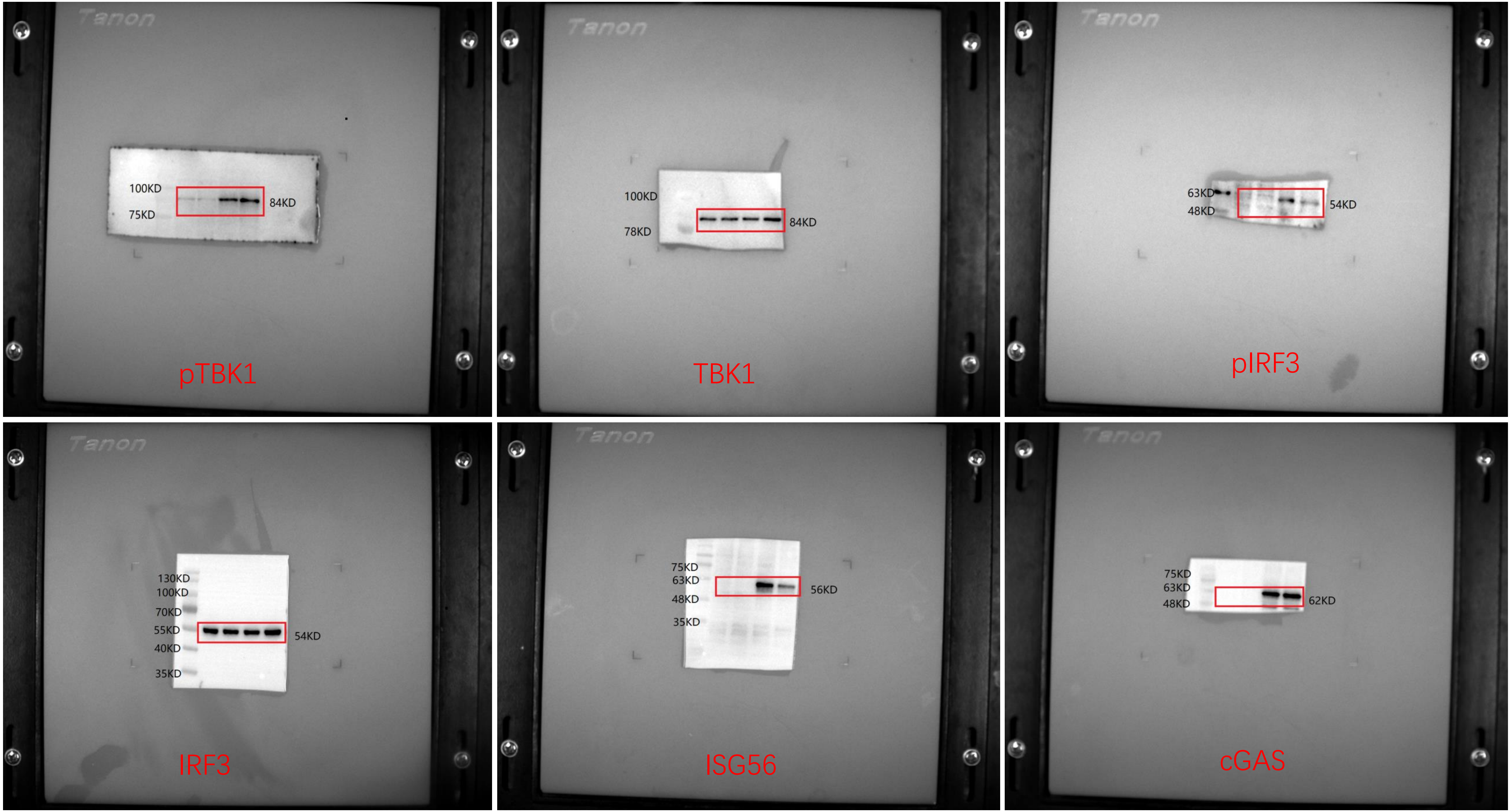

100KD
75KD
84KD
pTBK1
100KD
78KD
84KD
TBK1
63KD
48KD
54KD
pIRF3
130KD
100KD
70KD
55KD
40KD
35KD
54KD
IRF3
75KD
63KD
48KD
35KD
56KD
ISG56
75KD
63KD
48KD
62KD
cGAS

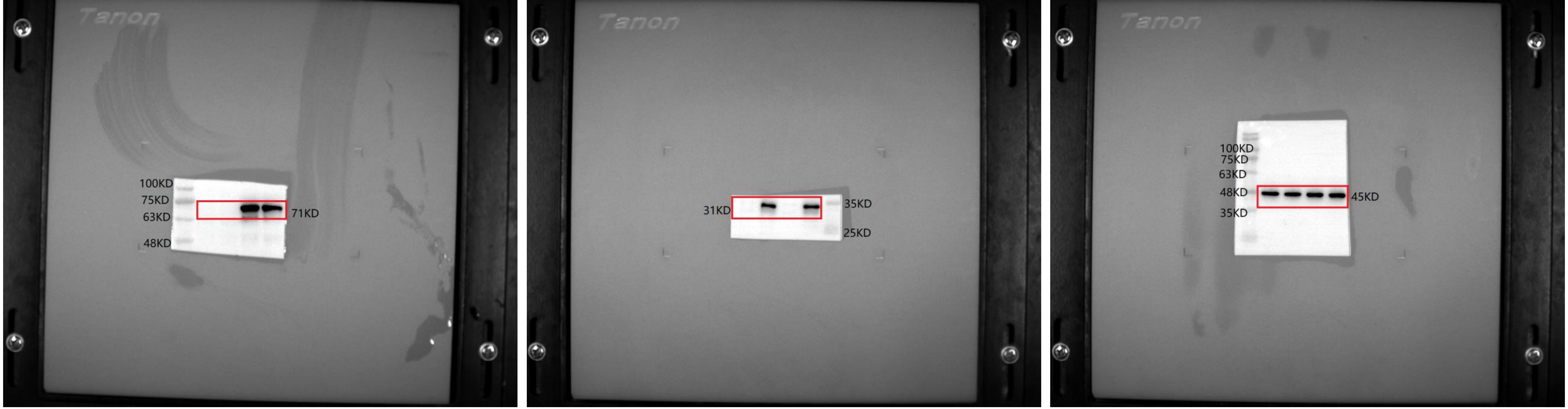
100KD
75KD
63KD
48KD
71KD
31KD
35KD
25KD
100KD
75KD
63KD
48KD
35KD
45KD

FIG2E

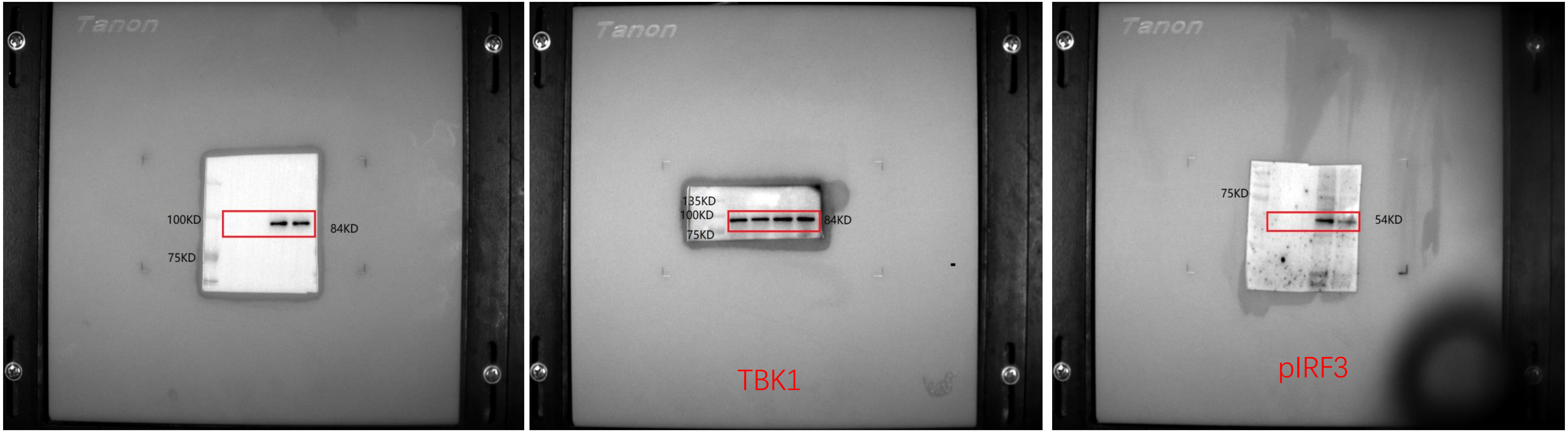
100KD
84KD
75KD
135KD
100KD
75KD
84KD
TBK1
75KD
54KD
pIRF3

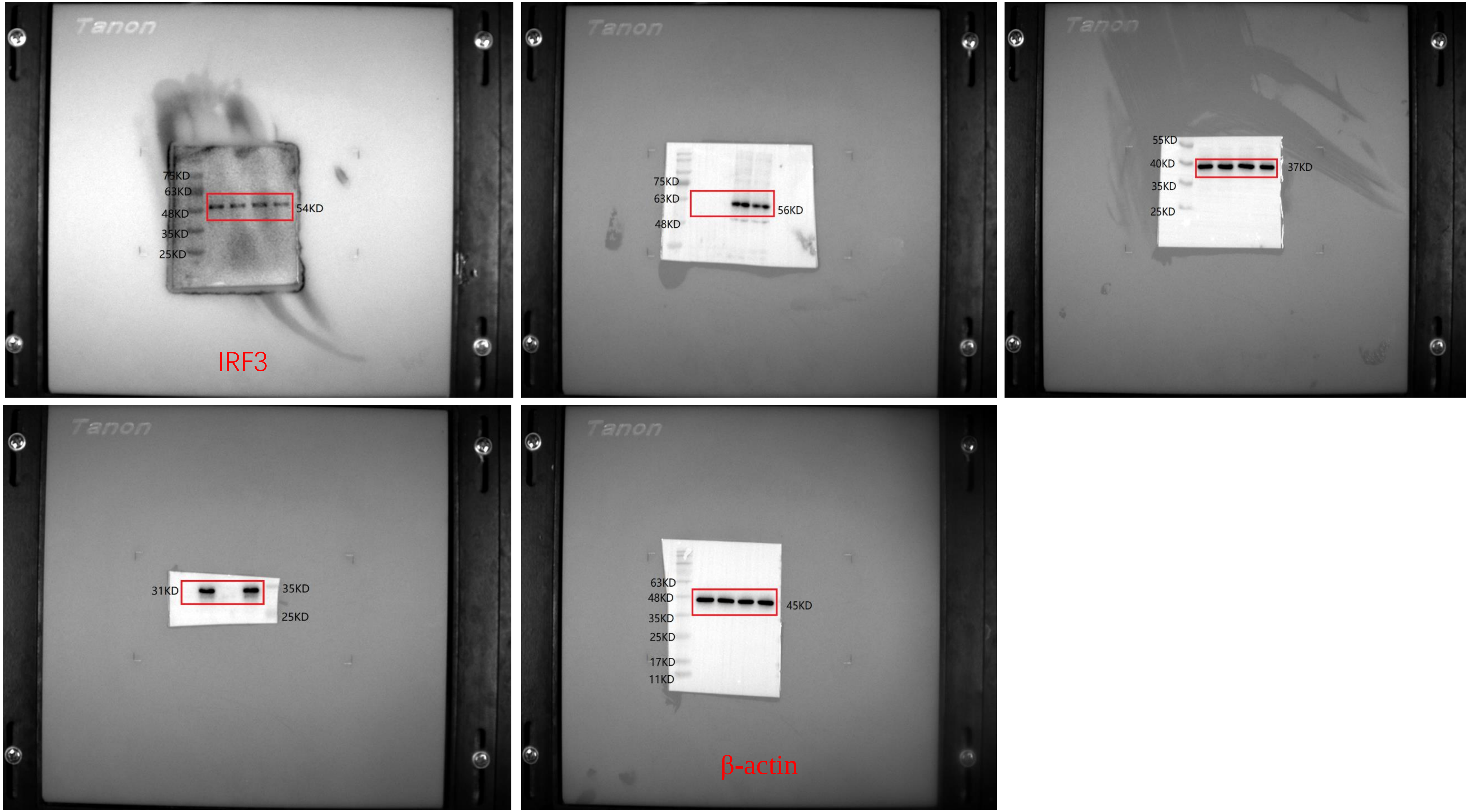
Tanon
75KD
63KD
48KD
35KD
25KD
54KD
IRF3
Tanon
75KD
63KD
48KD
56KD
Tanon
55KD
40KD
35KD
25KD
37KD
Tanon
31KD
35KD
25KD
Tanon
63KD
48KD
35KD
25KD
17KD
11KD
45KD
β-actin

FIG2F

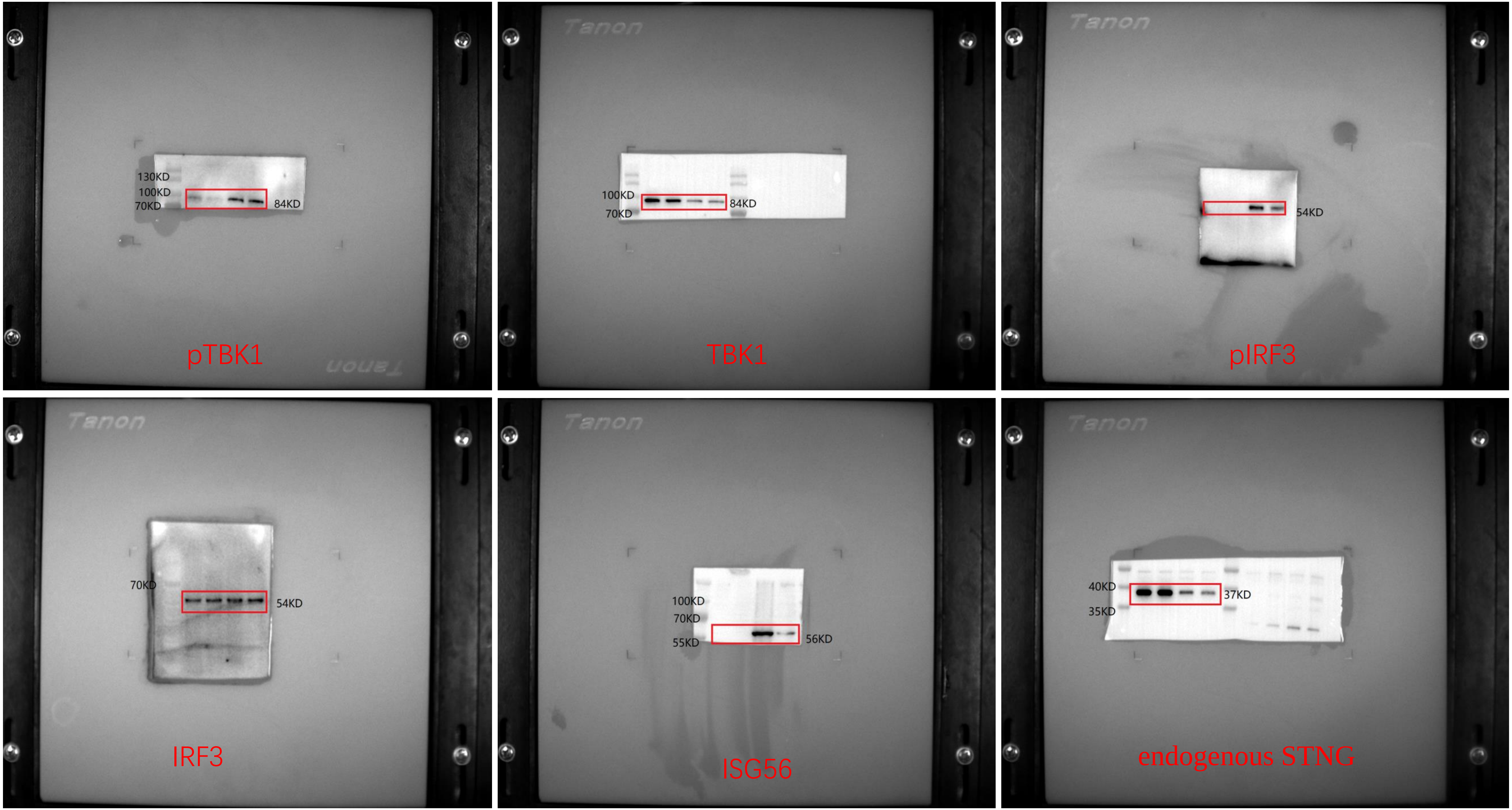

Tanon
130KD
100KD
70KD
84KD
pTBK1
100KD
70KD
84KD
TBK1
54KD
pIRF3
70KD
54KD
IRF3
100KD
70KD
55KD
56KD
ISG56
40KD
35KD
37KD
endogenous STNG

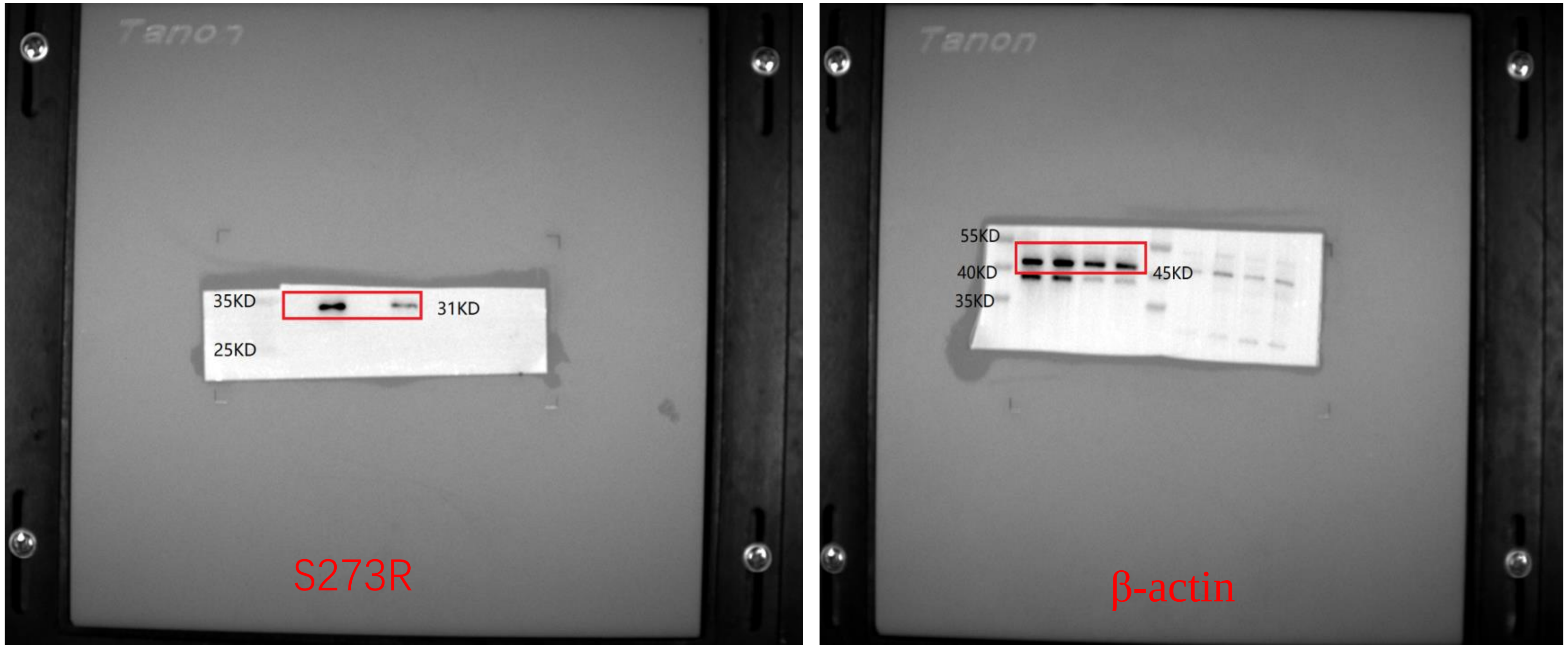
35KD
31KD
25KD
S273R
55KD
40KD
45KD
35KD
β-actin

FIG4B

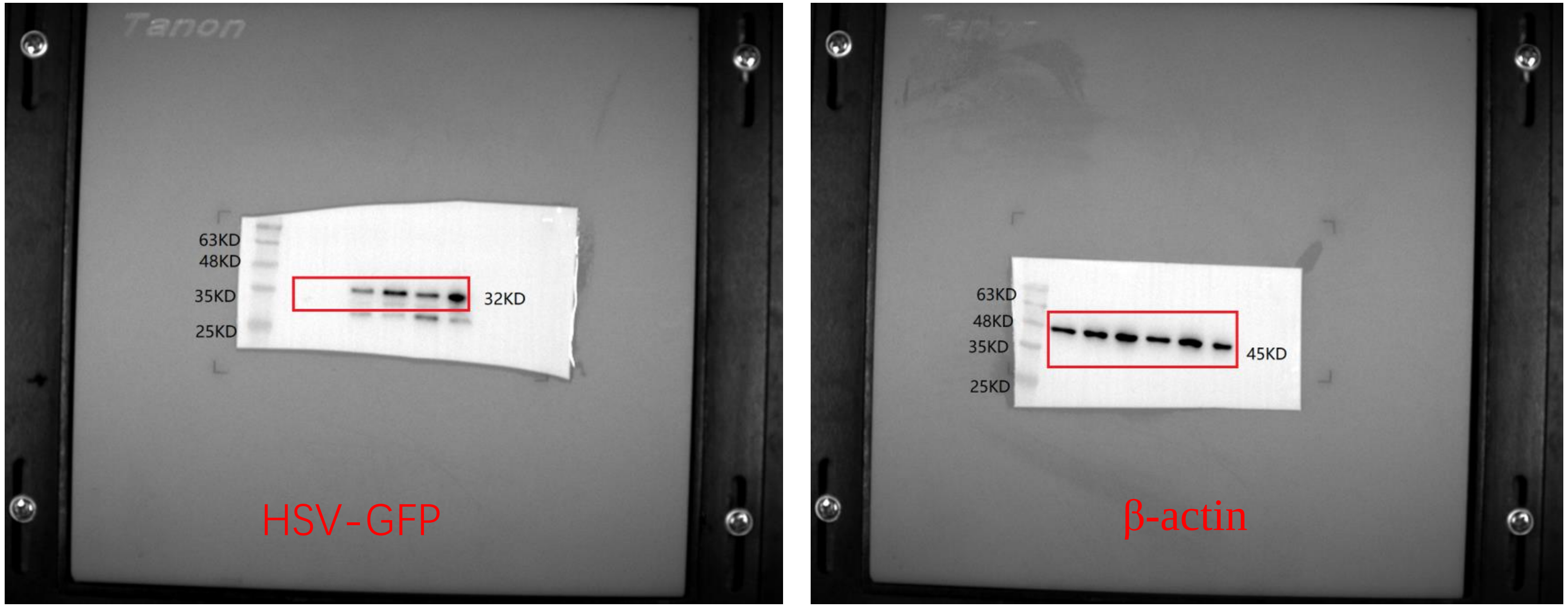
63KD
48KD
35KD
32KD
25KD
HSV-GFP
63KD
48KD
35KD
45KD
25KD
β-actin

FIG6G

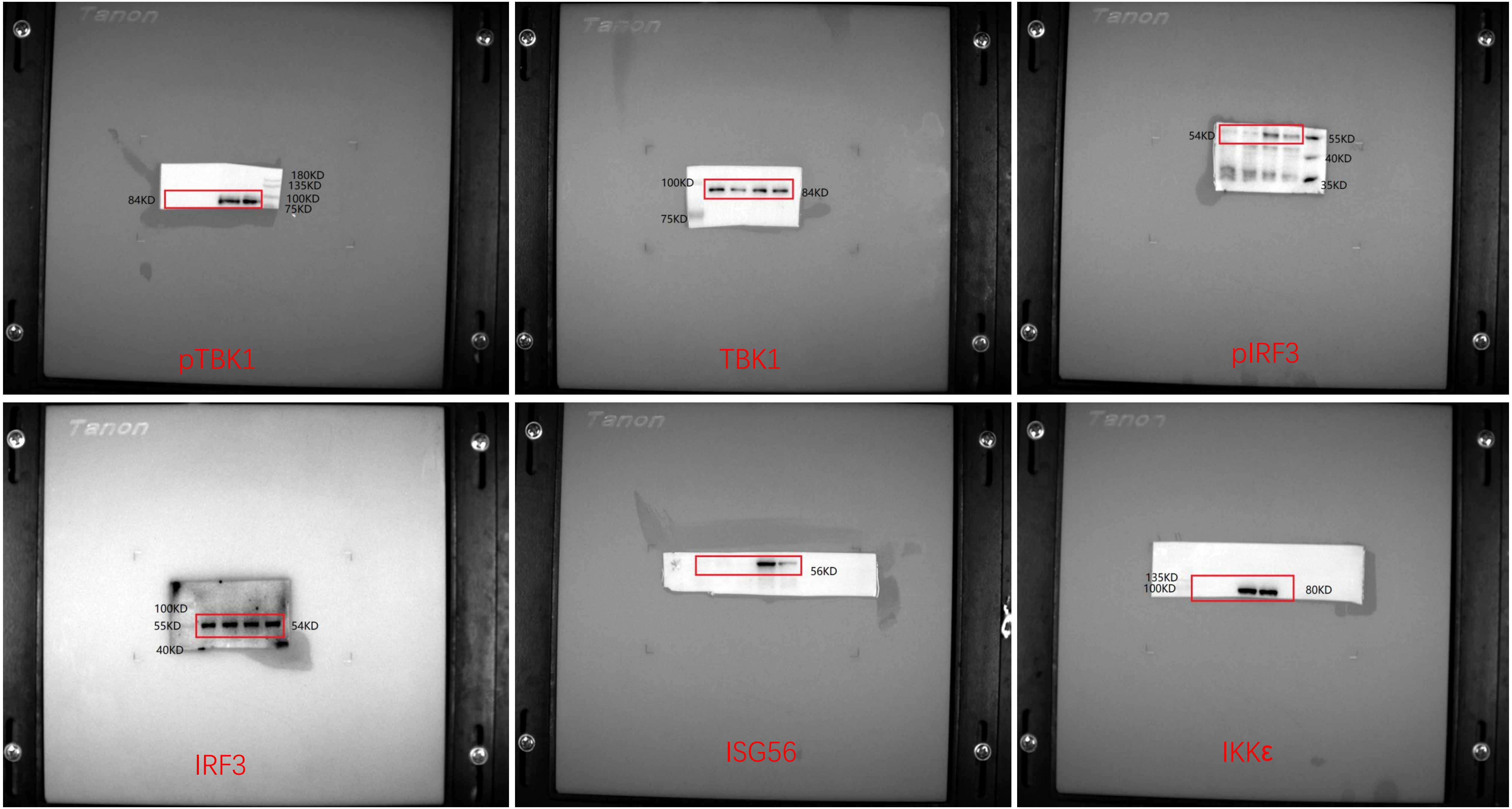

84KD
180KD
135KD
100KD
75KD
pTBK1
100KD
75KD
84KD
TBK1
54KD
55KD
40KD
35KD
pIRF3
100KD
55KD
40KD
54KD
IRF3
56KD
ISG56
135KD
100KD
80KD
IKKε

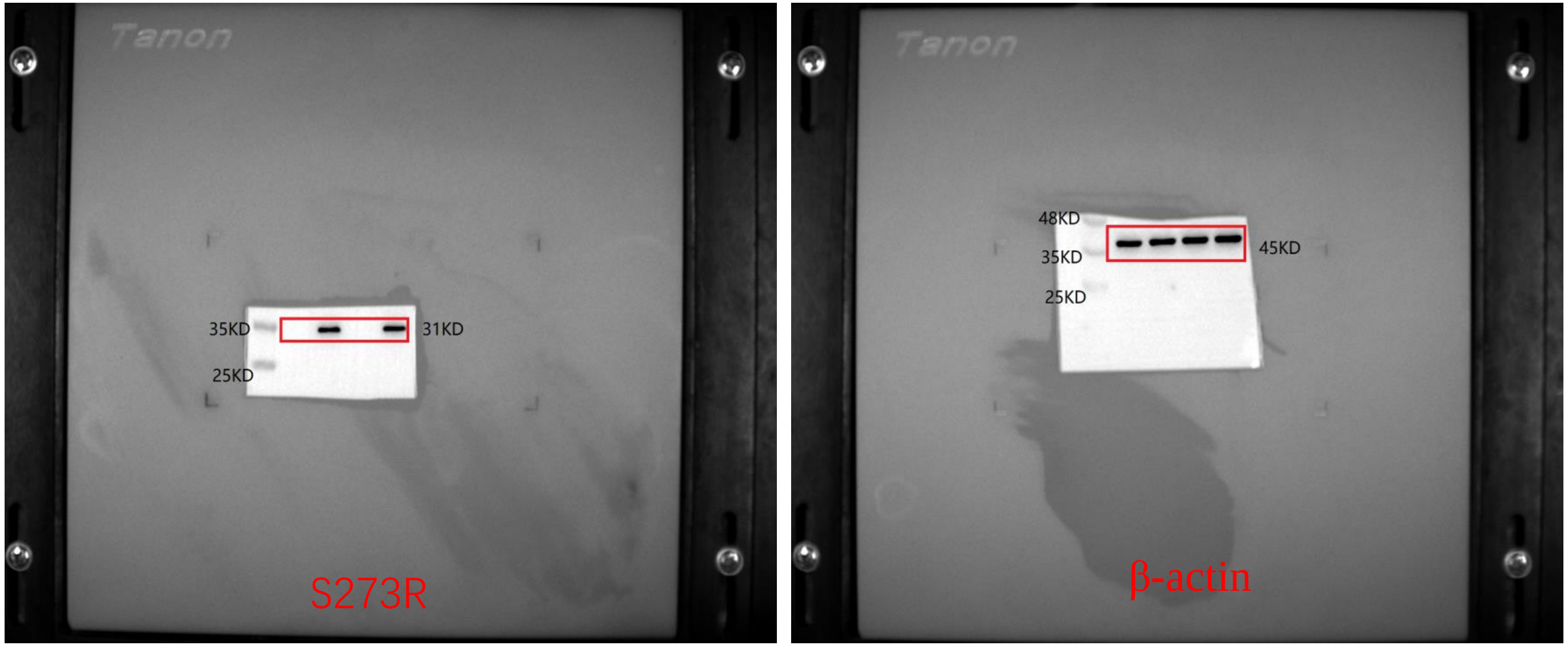
35KD
25KD
31KD
S273R
48KD
35KD
25KD
45KD
β-actin

FIG7A

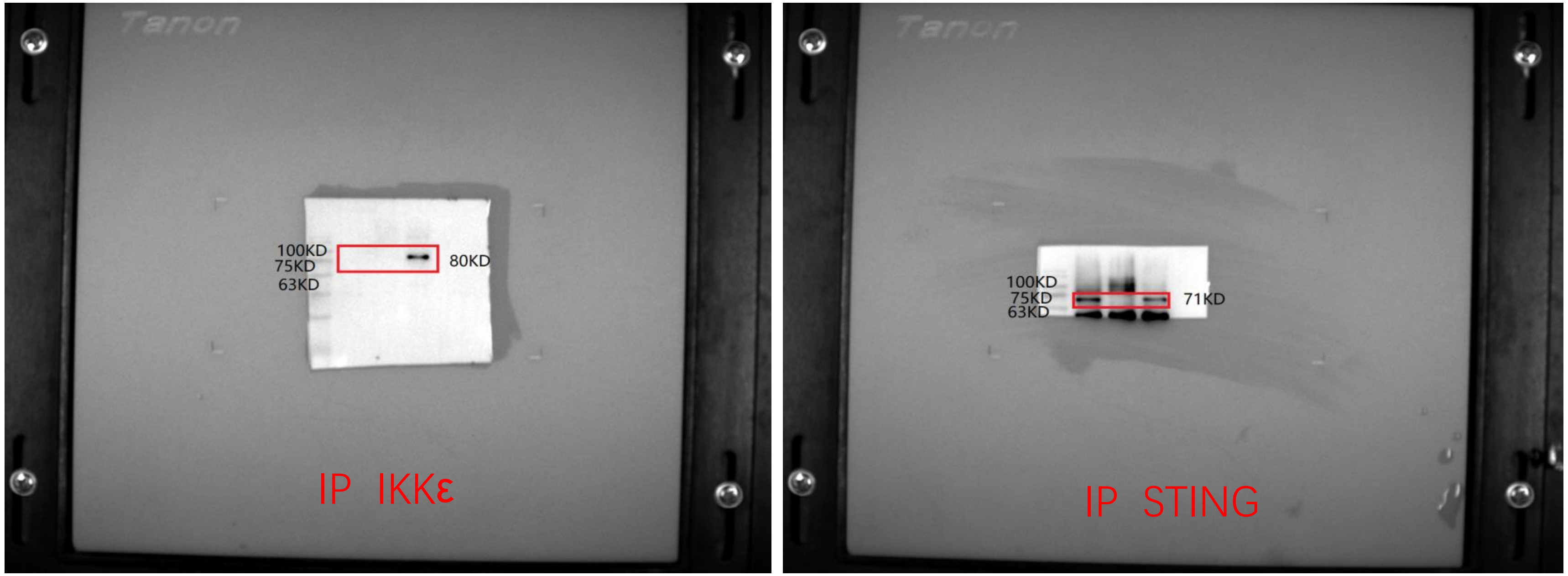
100KD
75KD
63KD
80KD
IP IKKε
100KD
75KD
63KD
71KD
IP STING

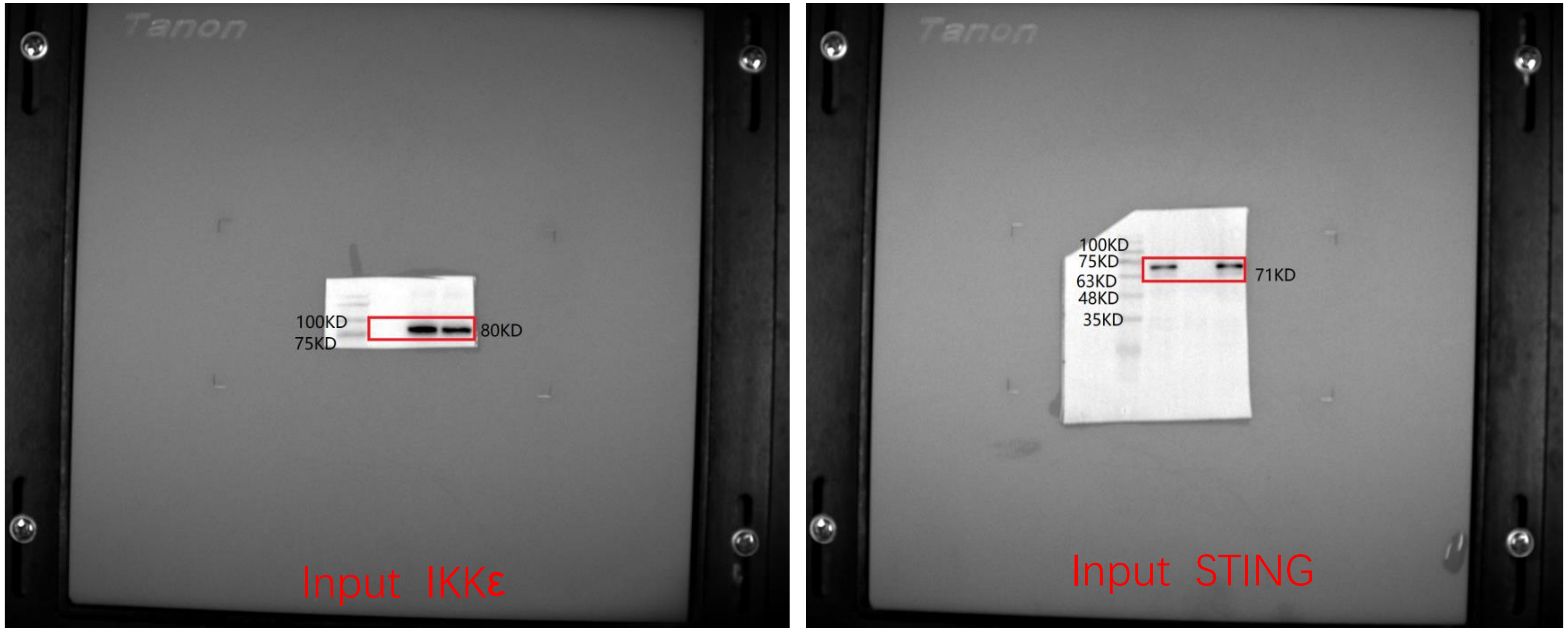
Tanon
100KD
75KD
80KD
Input IKKε
Tanon
100KD
75KD
63KD
48KD
35KD
71KD
Input STING

FIG7B

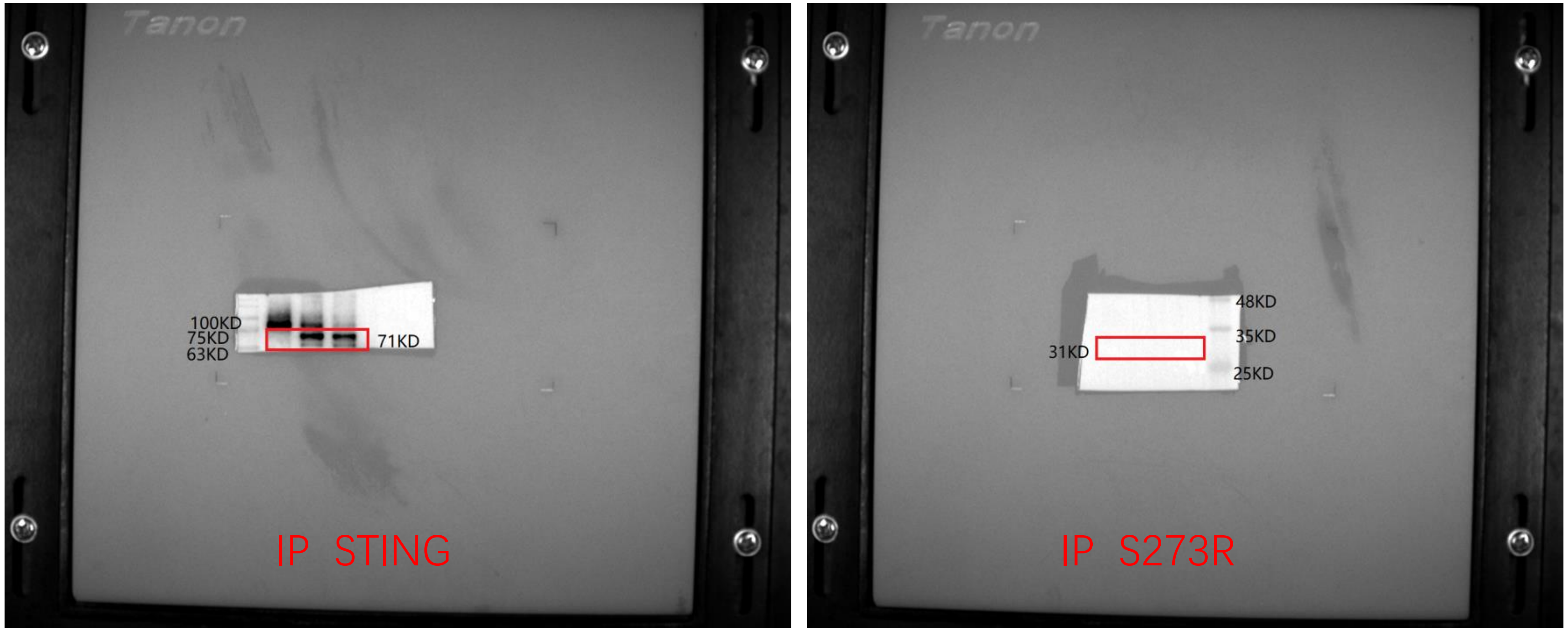
Tanon
100KD
75KD
63KD
71KD
IP STING
Tanon
48KD
35KD
31KD
25KD
IP S273R

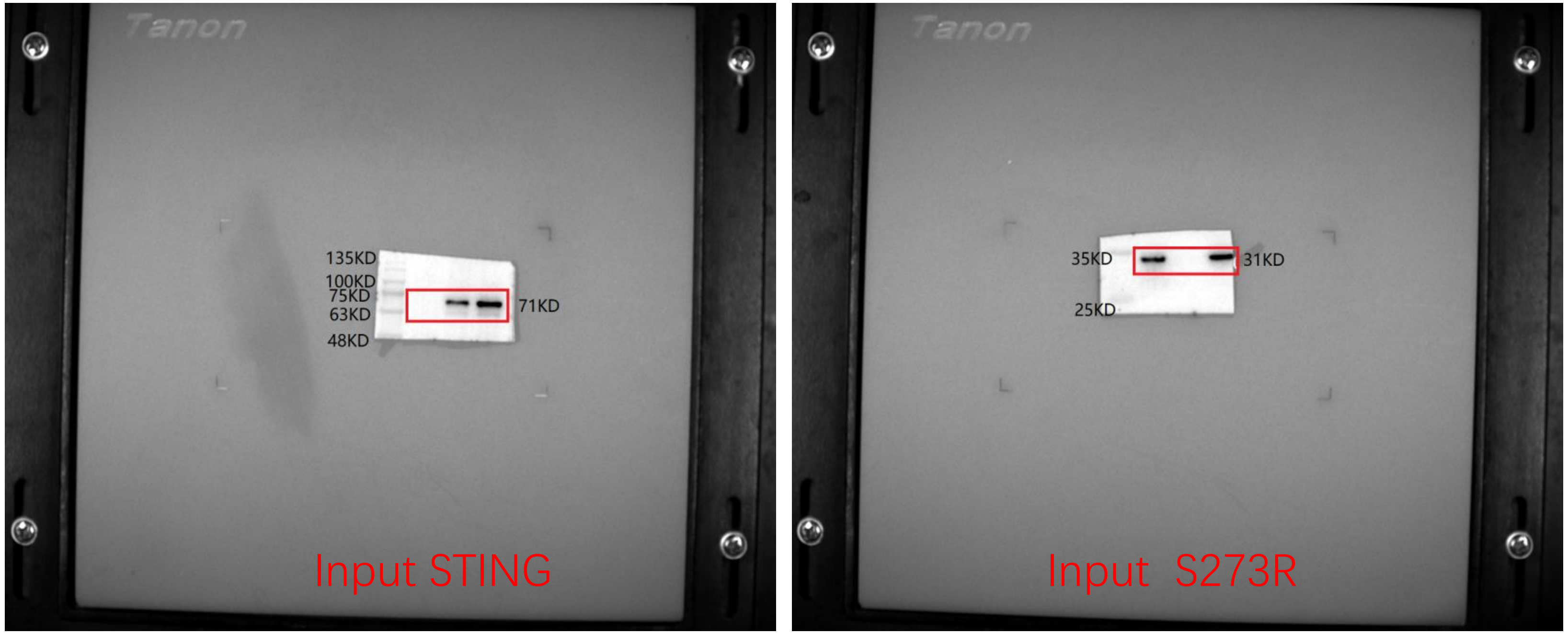
135KD
100KD
75KD
63KD
48KD
71KD
Input STING
35KD
31KD
25KD
Input S273R

FIG7C

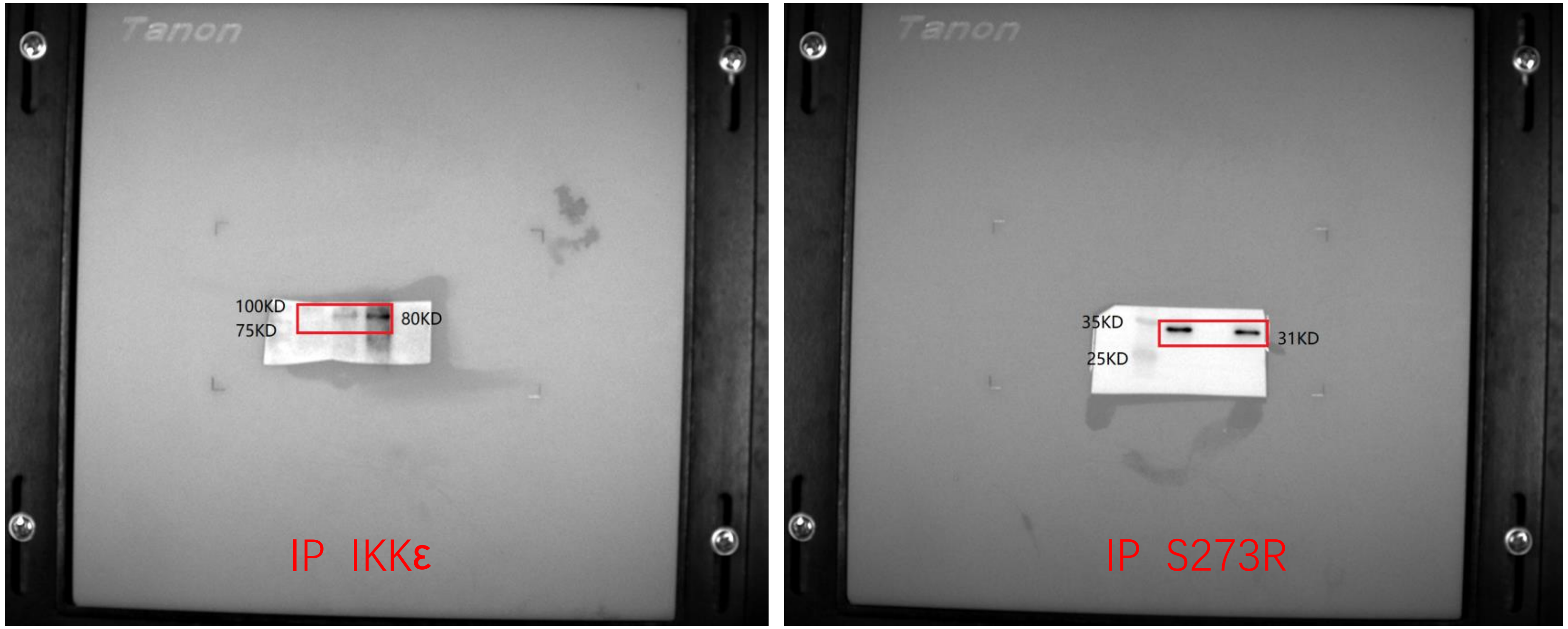
100KD
75KD
80KD
IP IKKε
35KD
31KD
25KD
IP S273R

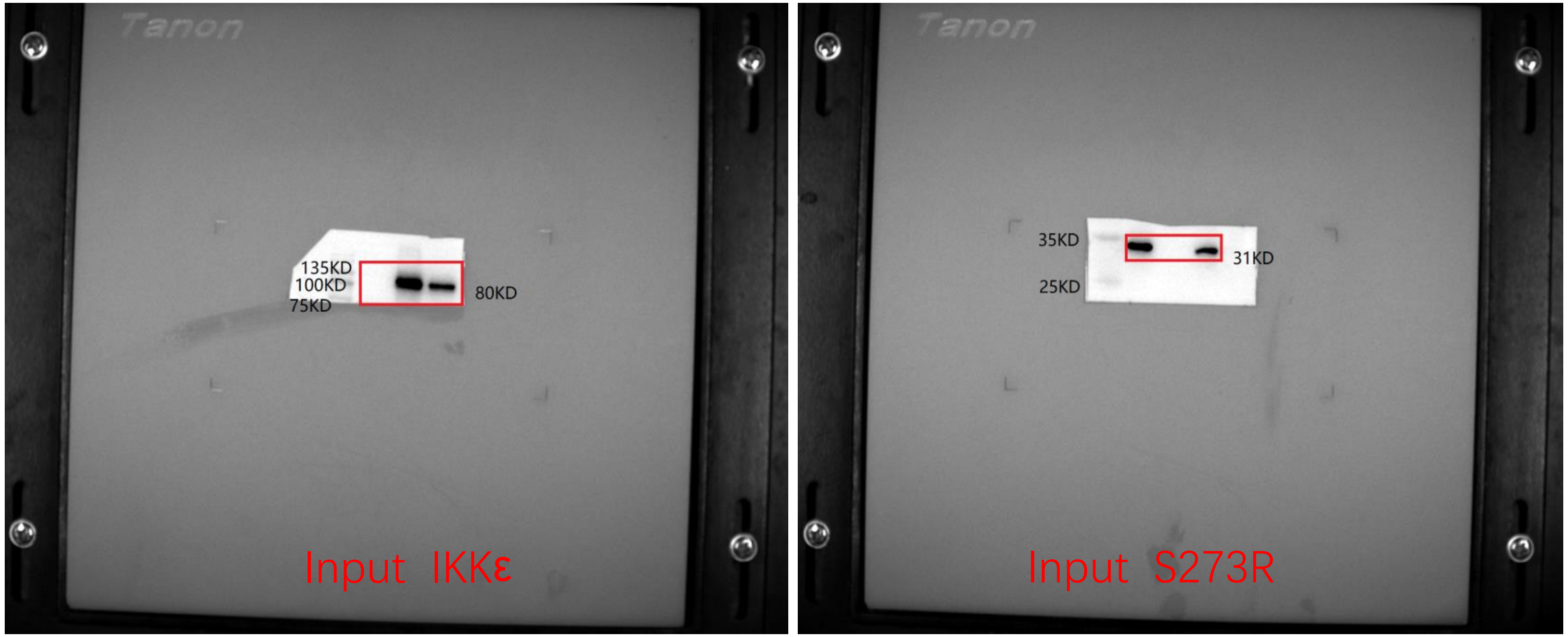
135KD
100KD
75KD
80KD
Input IKKε
35KD
25KD
31KD
Input S273R

FIG7D

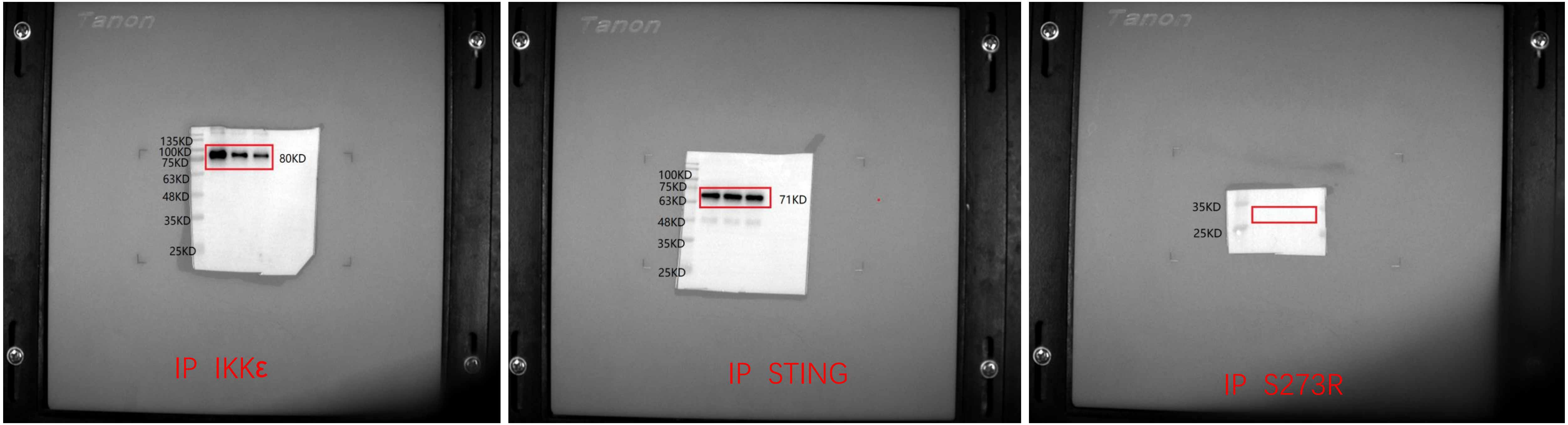
135KD
100KD
75KD
63KD
48KD
35KD
25KD
80KD
IP IKKε
100KD
75KD
63KD
48KD
35KD
25KD
71KD
IP STING
35KD
25KD
IP S273R

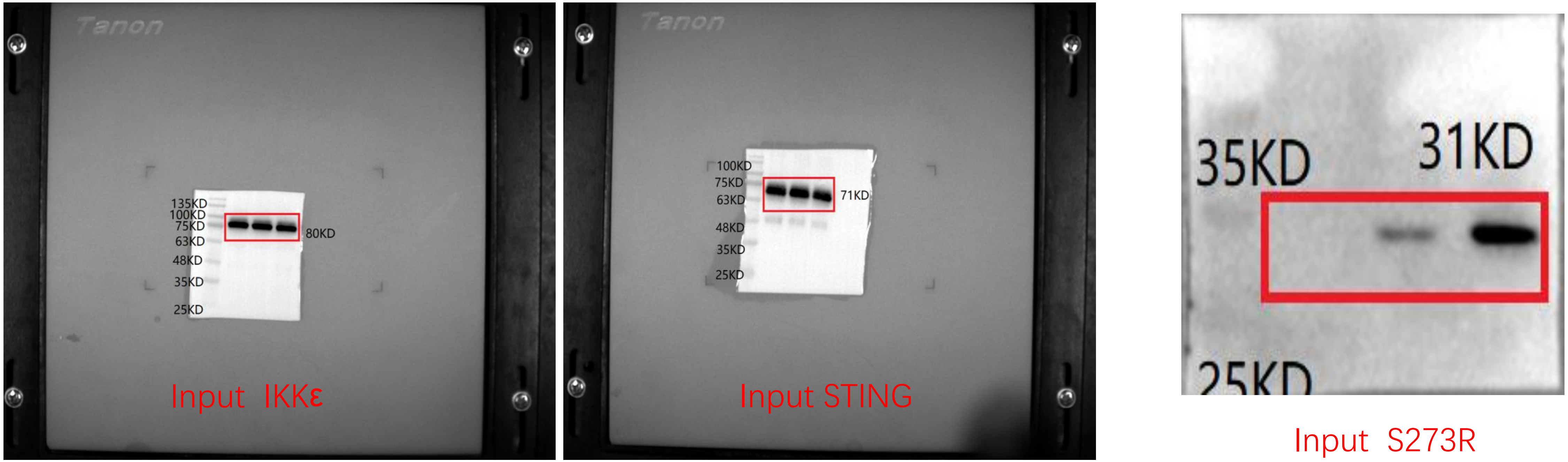

Input S273R

FIG8A

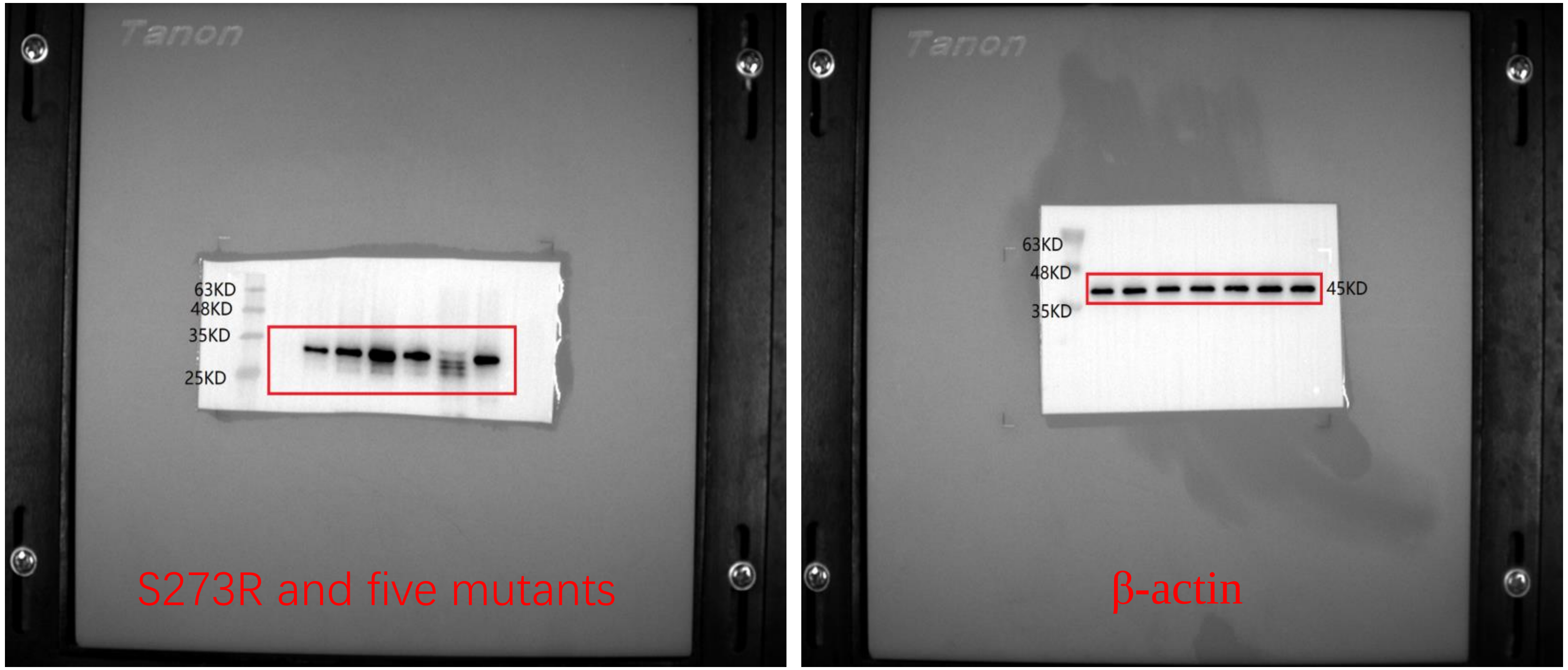

FIG8D

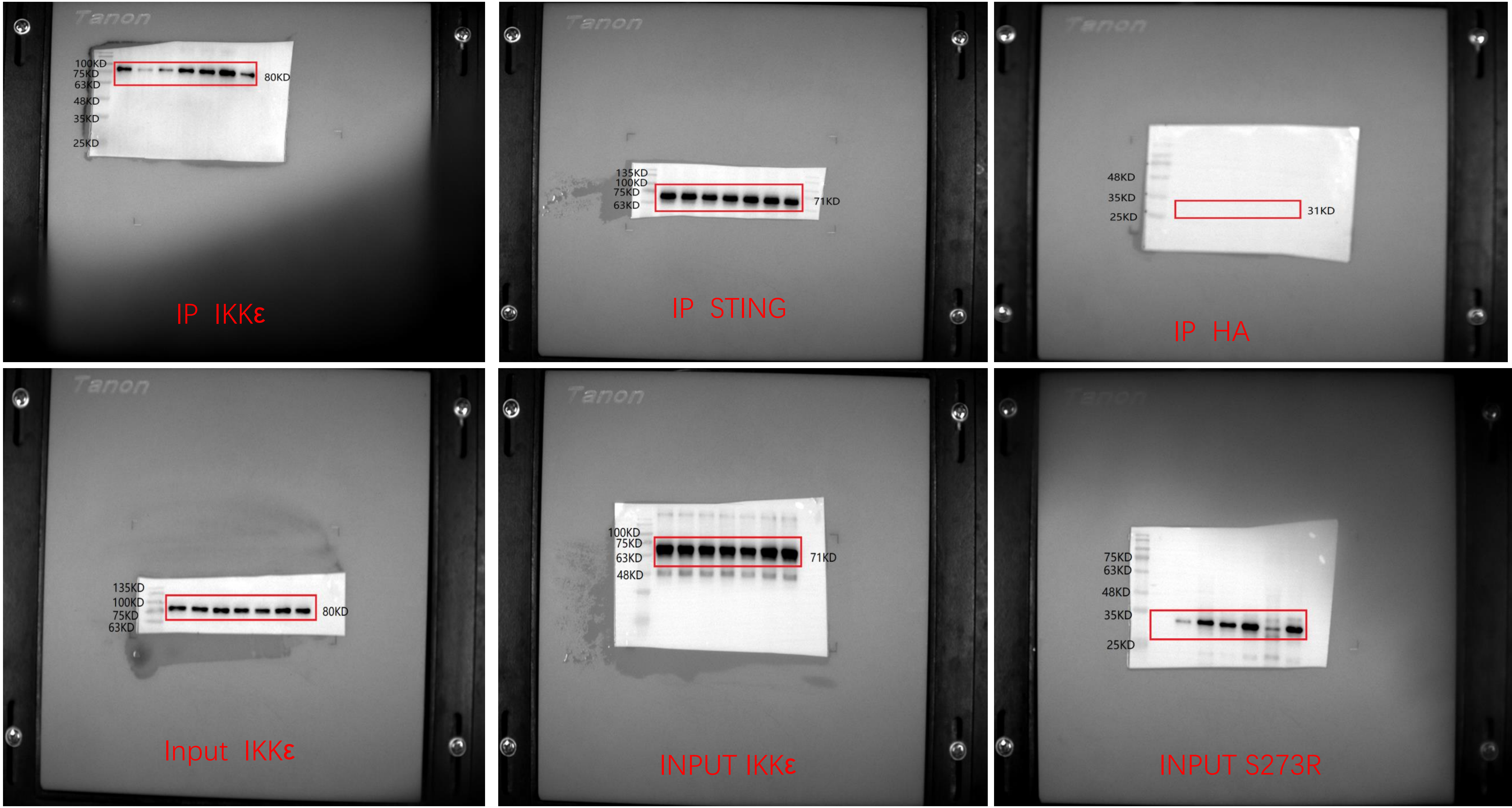

Tanon
100KD
75KD
63KD
48KD
35KD
25KD
80KD
IP IKKε
Tanon
135KD
100KD
75KD
63KD
71KD
IP STING
Tanon
48KD
35KD
25KD
31KD
IP HA
Tanon
135KD
100KD
75KD
63KD
80KD
Input IKKε
Tanon
100KD
75KD
63KD
48KD
71KD
INPUT IKKε
75KD
63KD
48KD
35KD
25KD
INPUT S273R

FIG9C

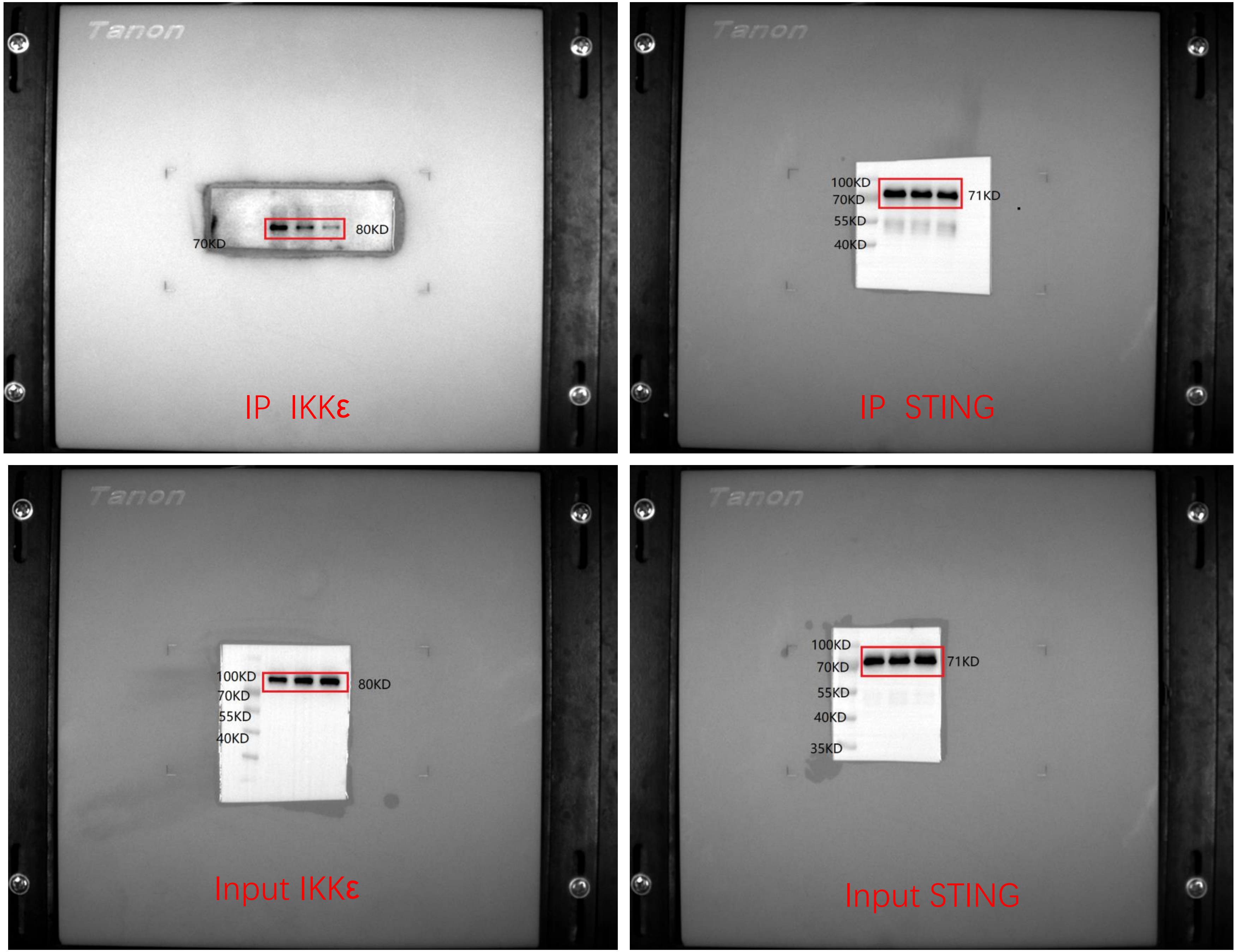
Tanon
70KD
80KD
IP IKKε
Tanon
100KD
70KD
55KD
40KD
71KD
IP STING
Tanon
100KD
70KD
55KD
40KD
80KD
Input IKKε
Tanon
100KD
70KD
55KD
40KD
35KD
71KD
Input STING

FIG9E

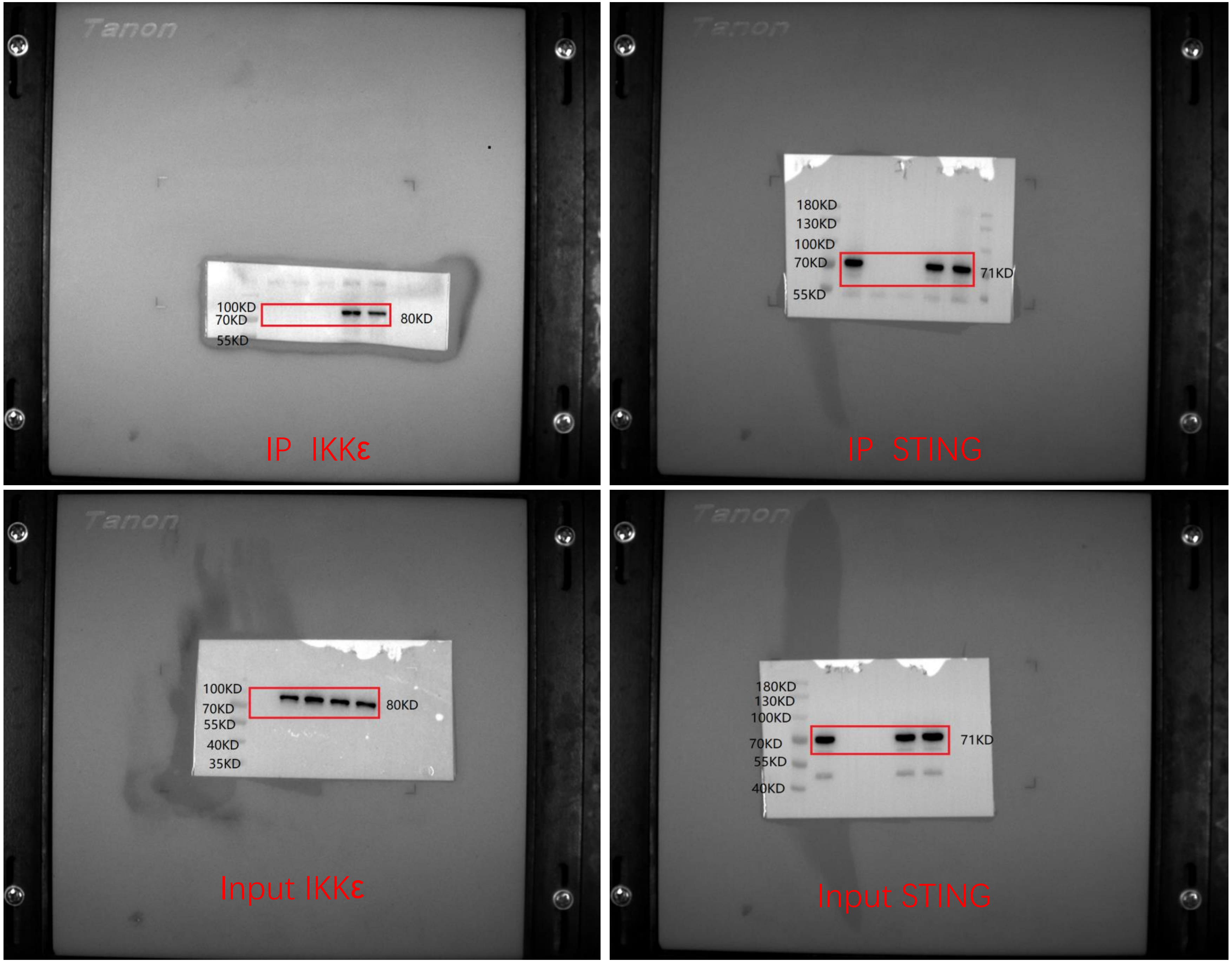
Tanon
100KD
70KD
55KD
80KD
IP IKKε
180KD
130KD
100KD
70KD
55KD
71KD
IP STING
100KD
70KD
55KD
40KD
35KD
80KD
Input IKKε
180KD
130KD
100KD
70KD
55KD
40KD
71KD
Input STING

FIG3A

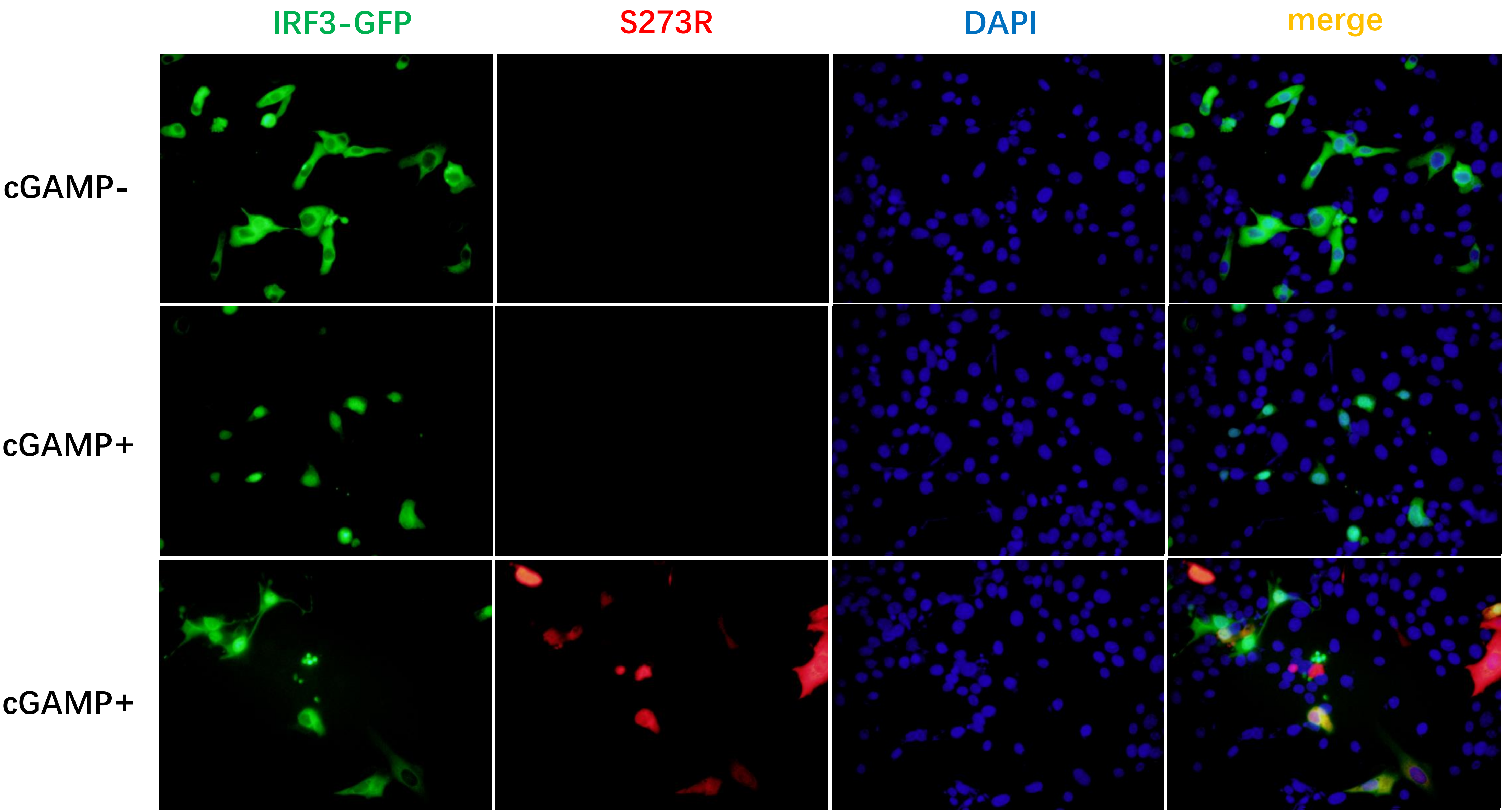

FIG3B

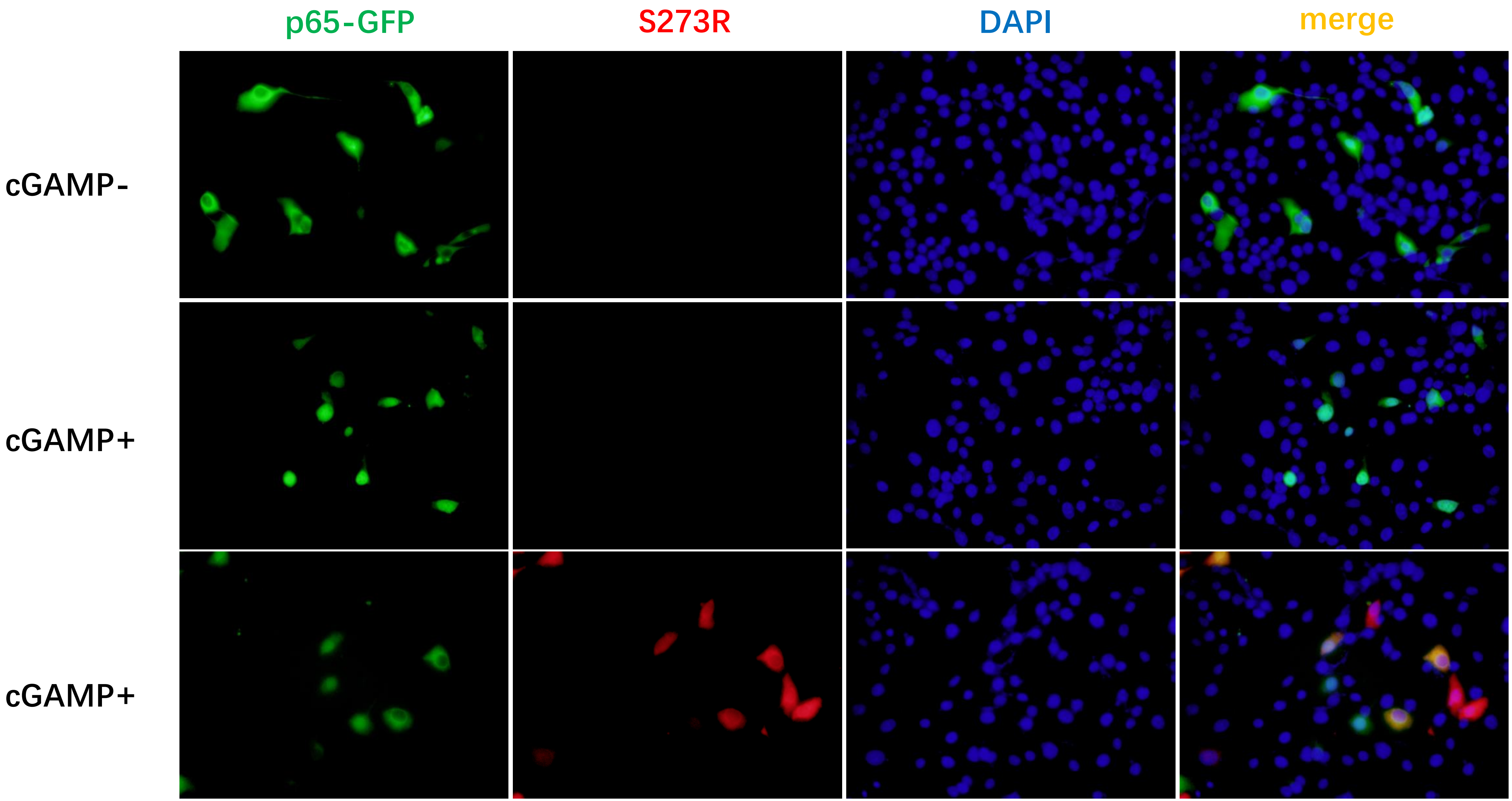

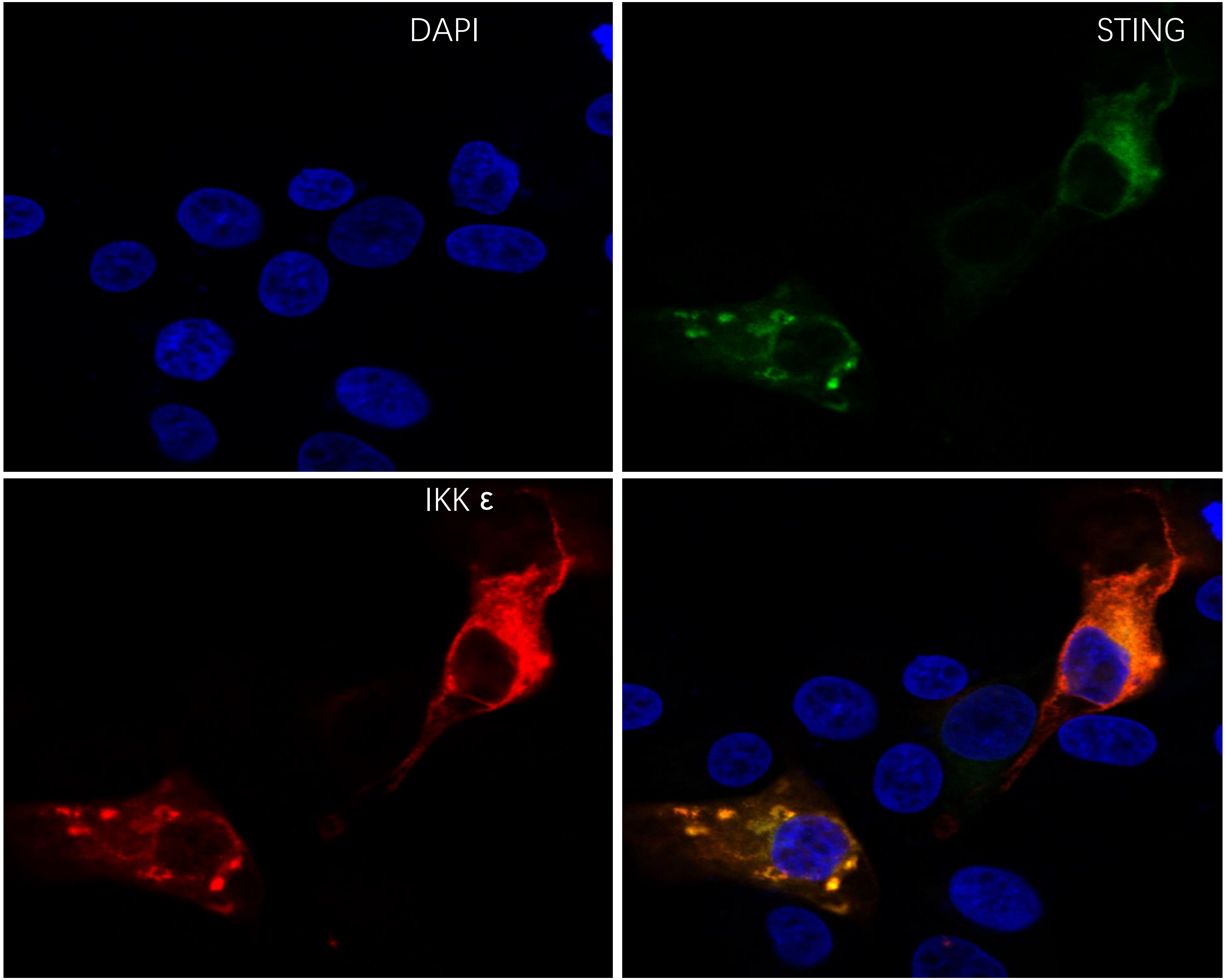

FIG7E

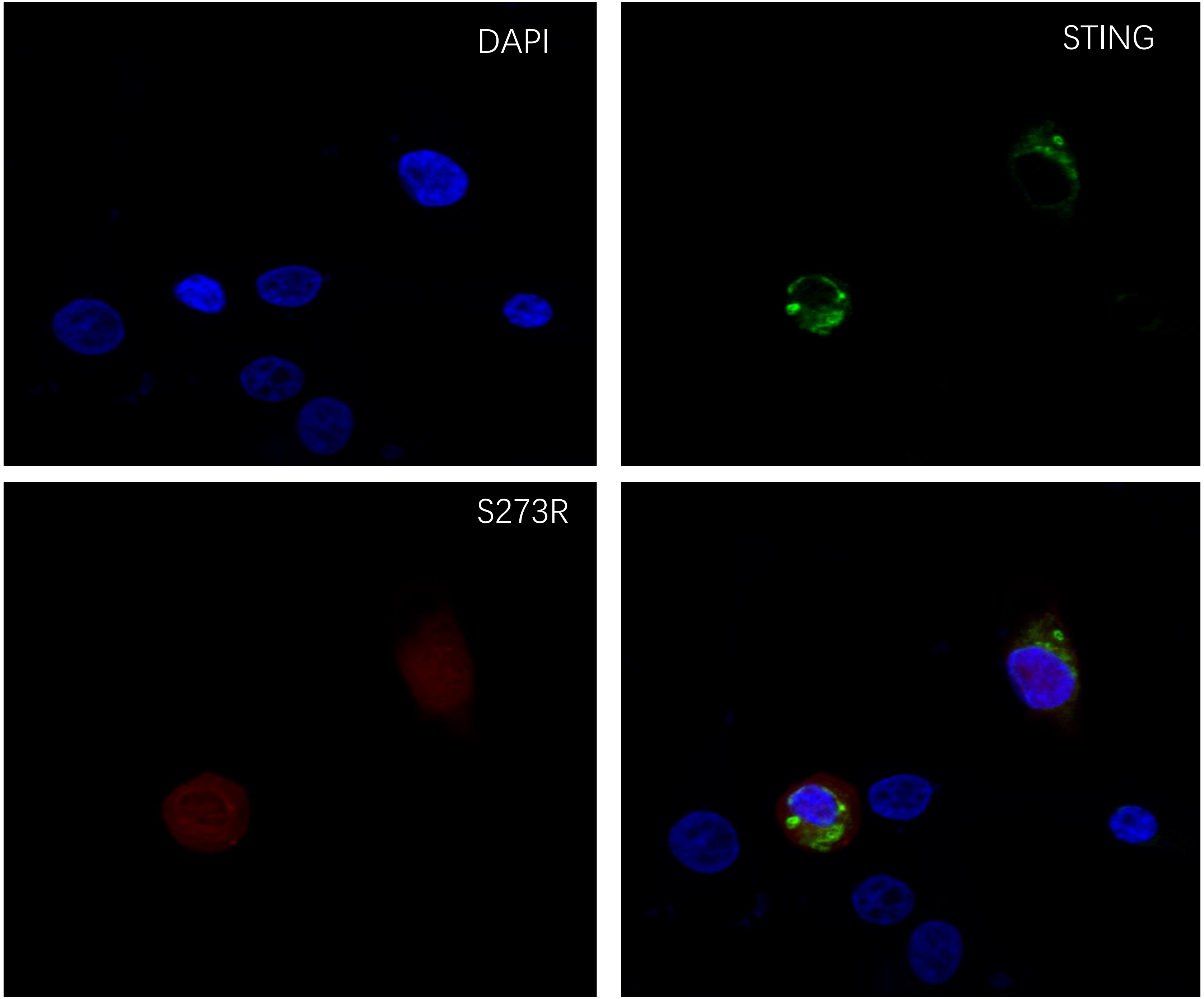

FIG7E

FIG9C

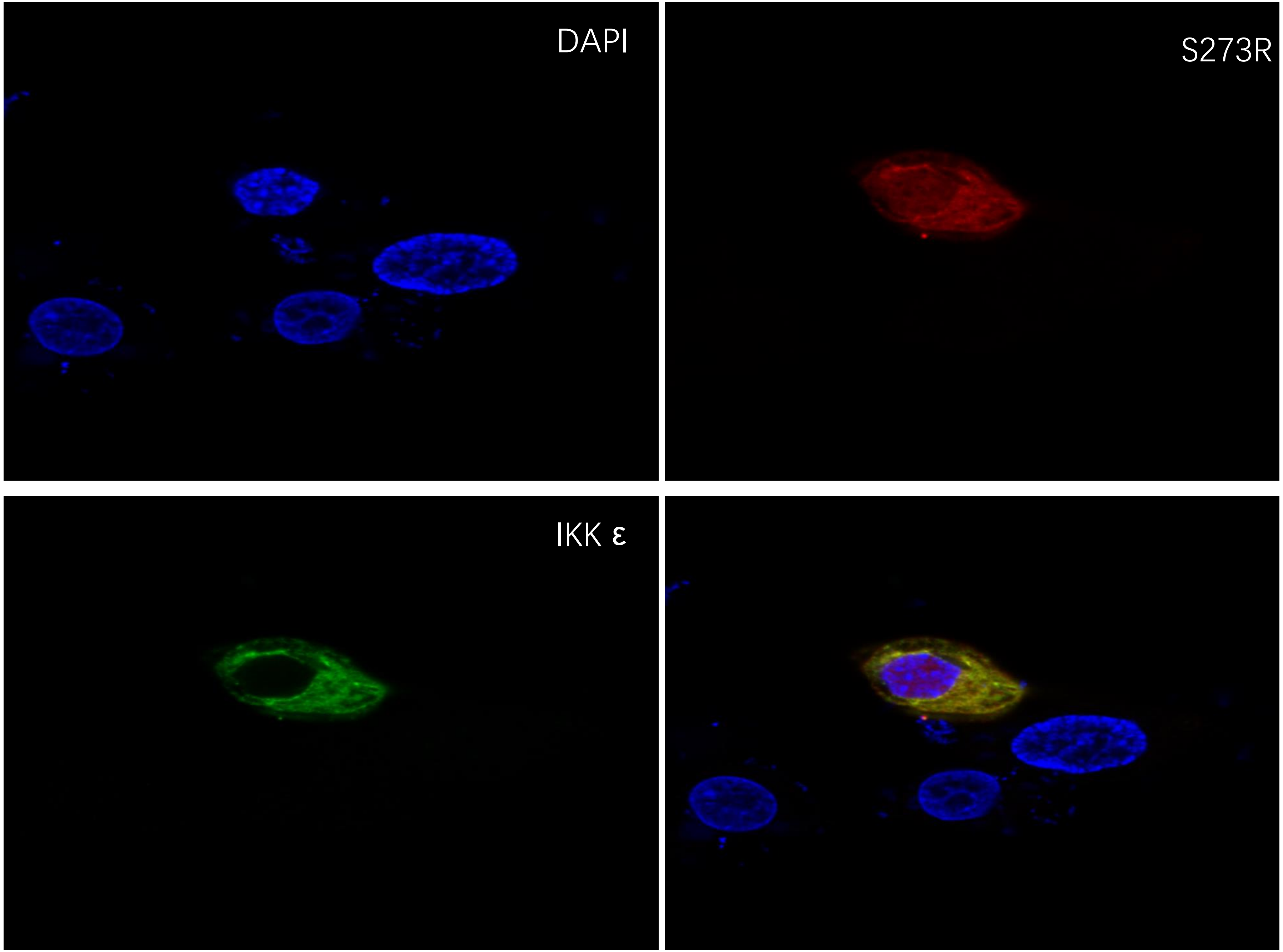

FIG7E

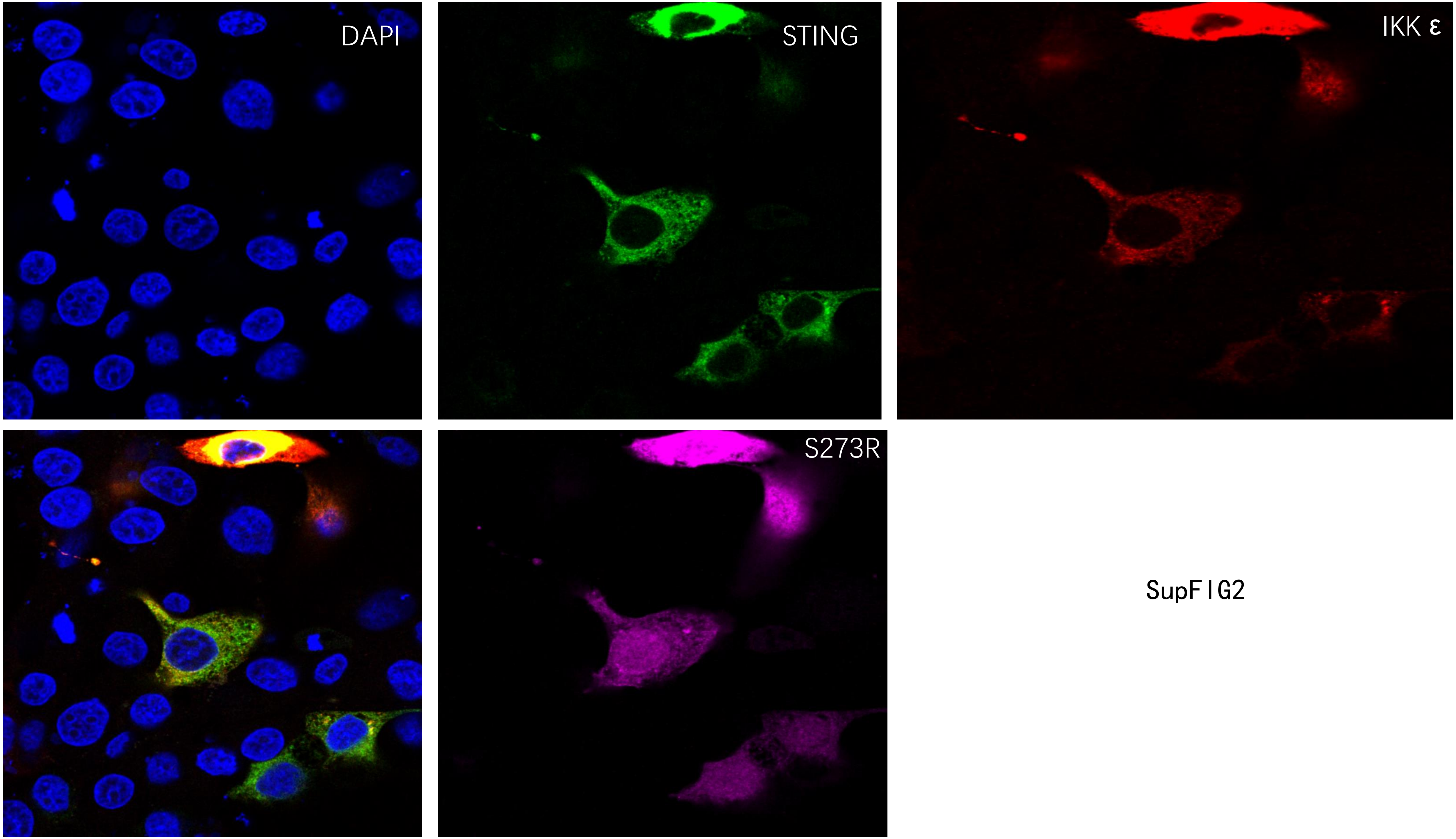

SupFIG2

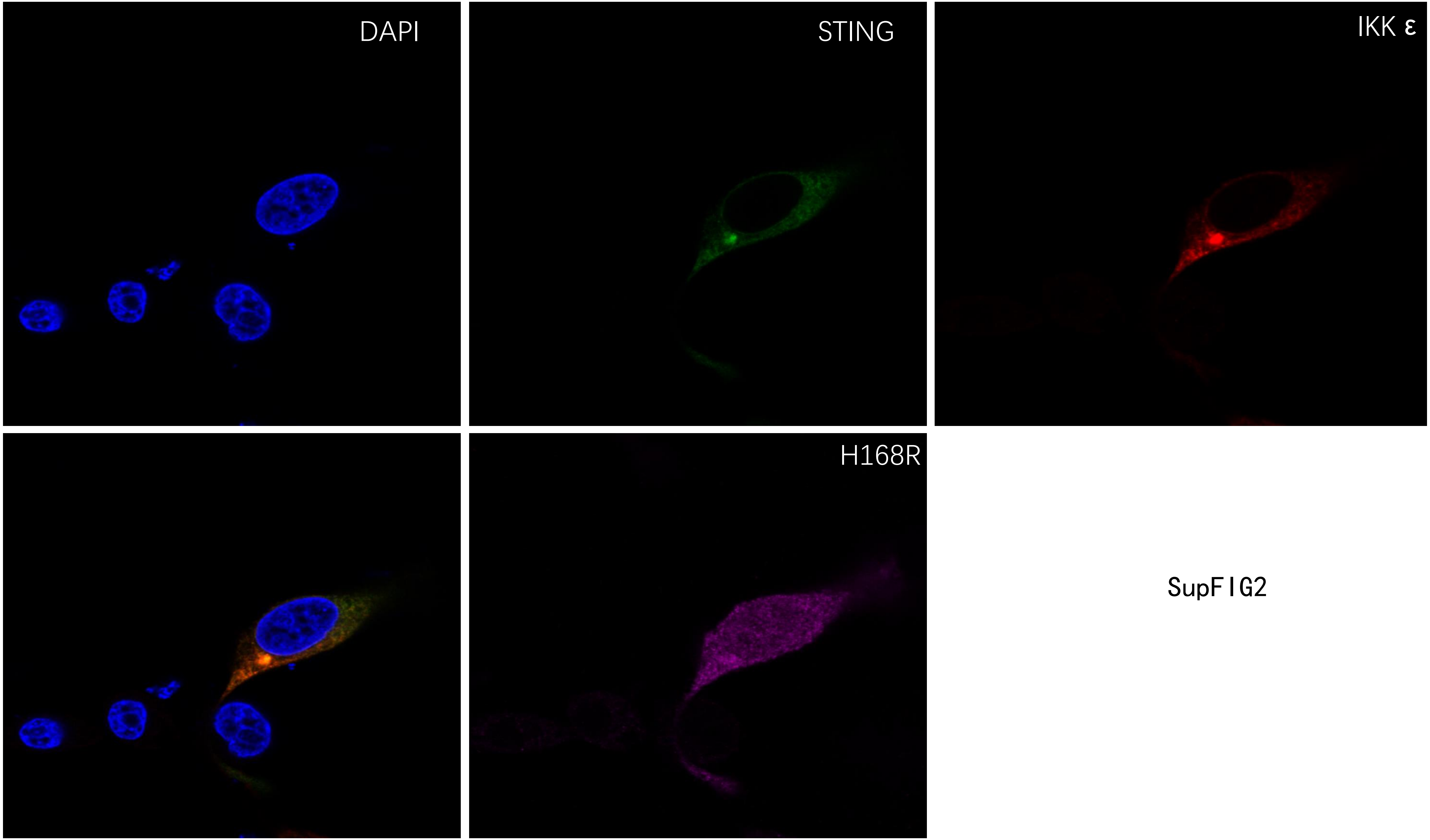

SupFIG2

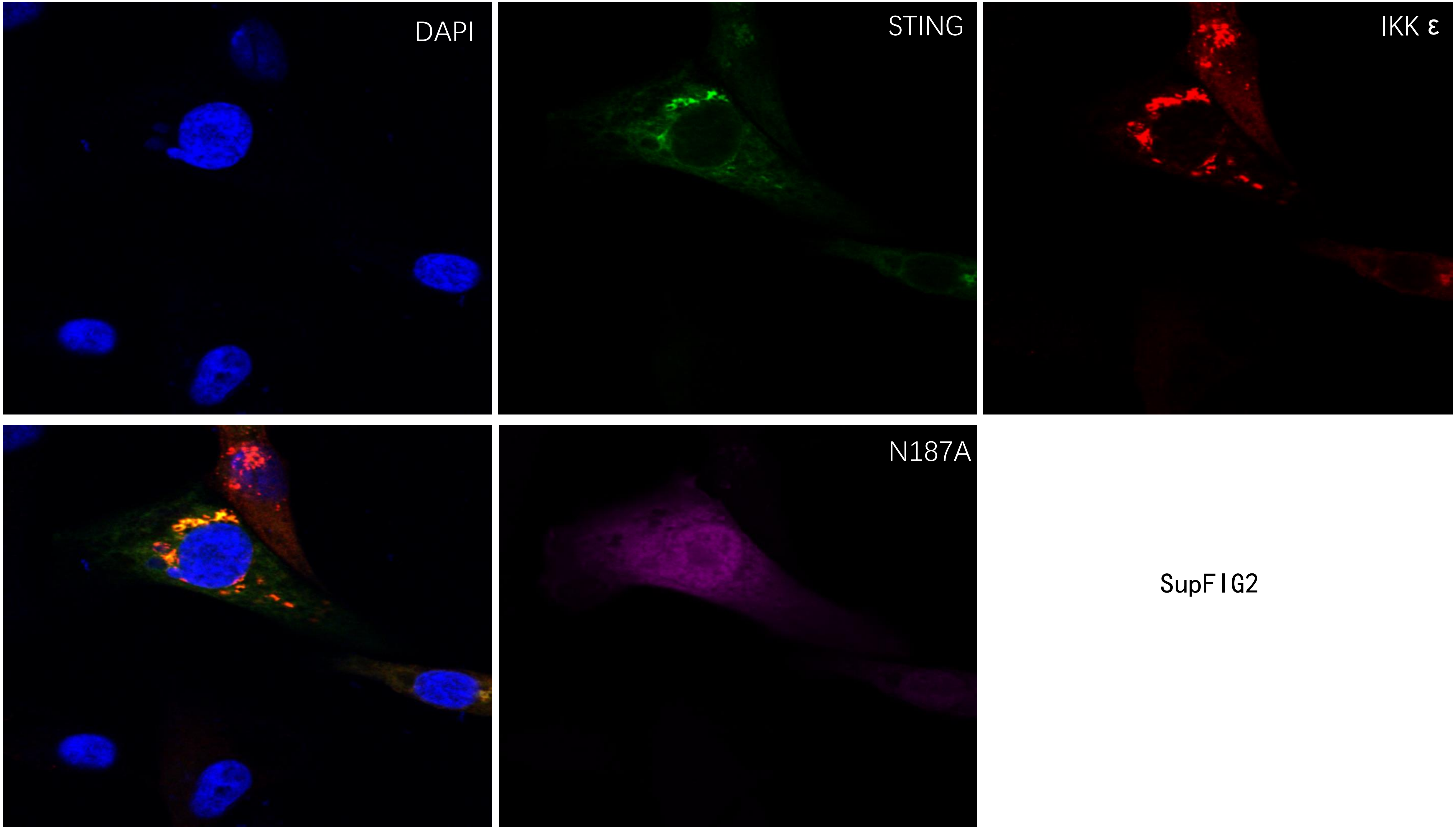

SupFIG2

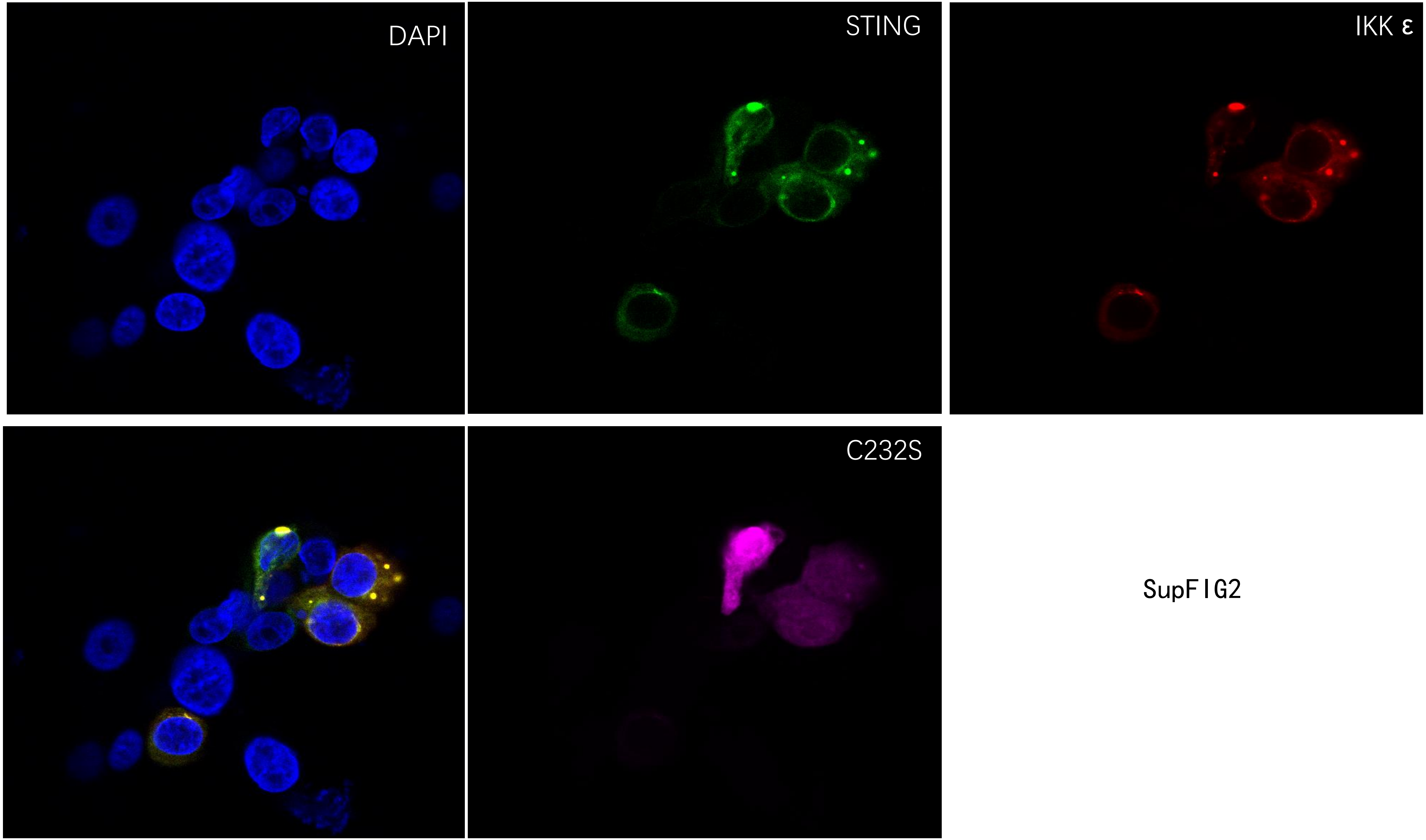

SupFIG2

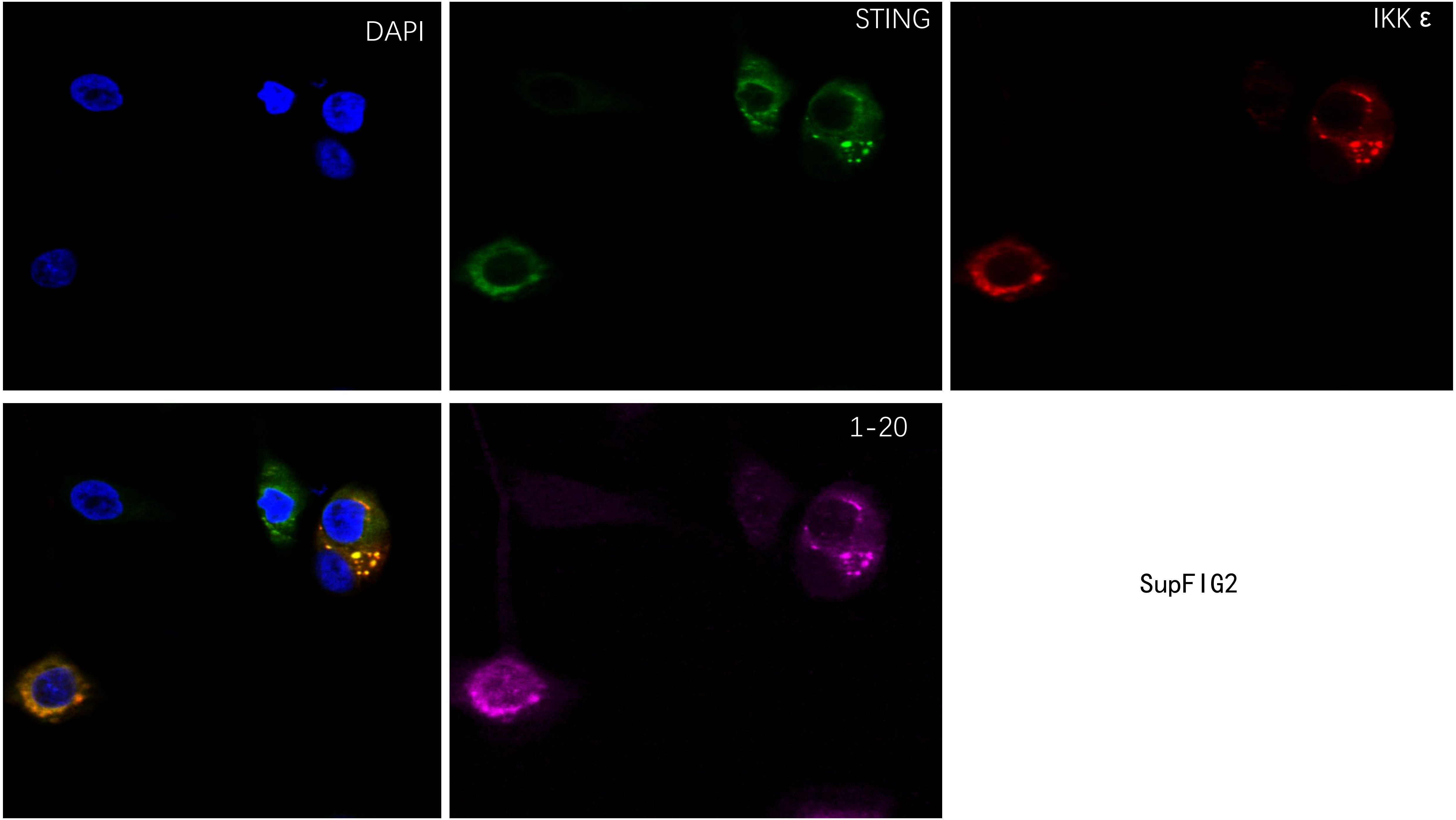

SupFIG2

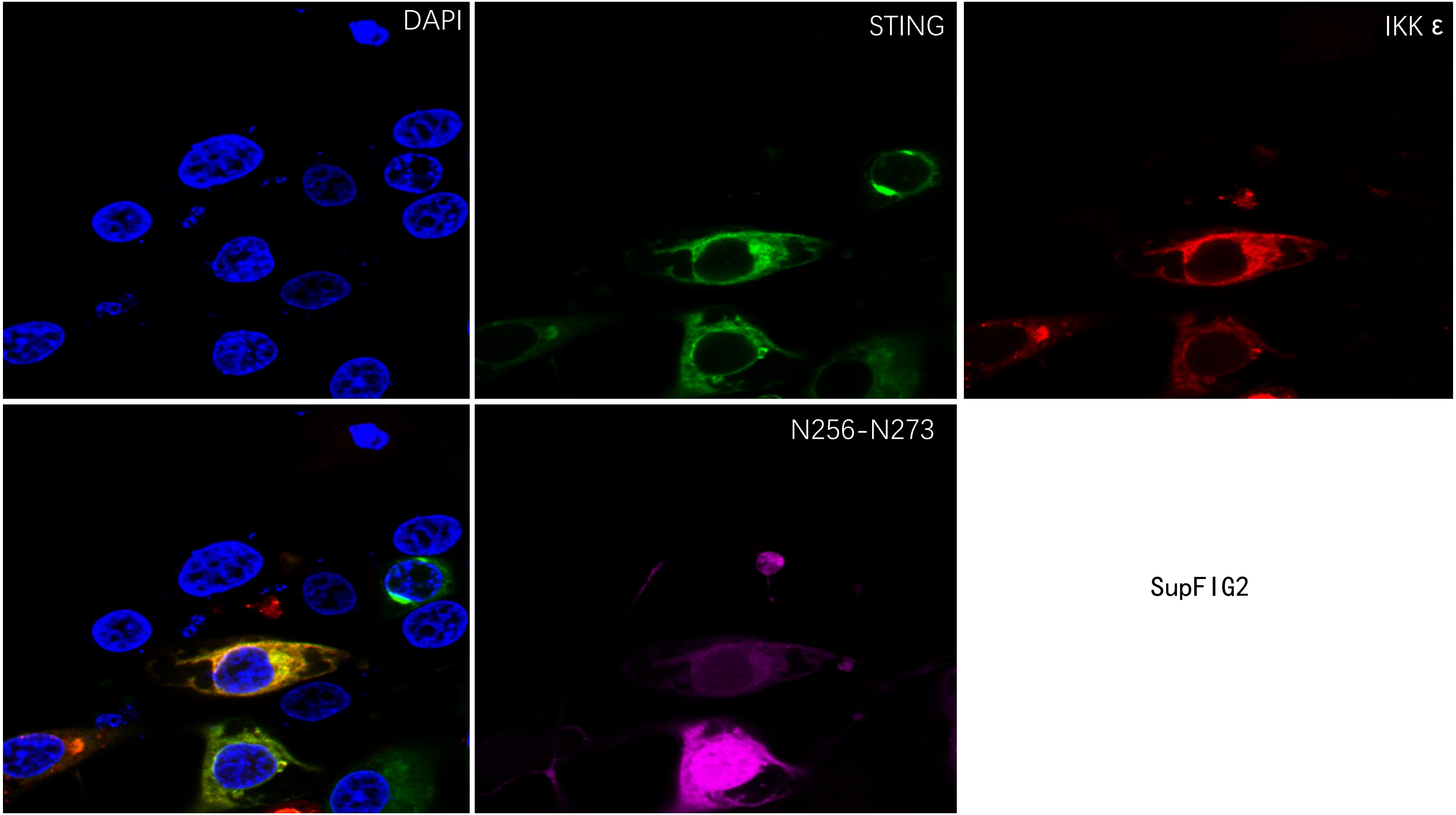

SupFIG2

PAM cell

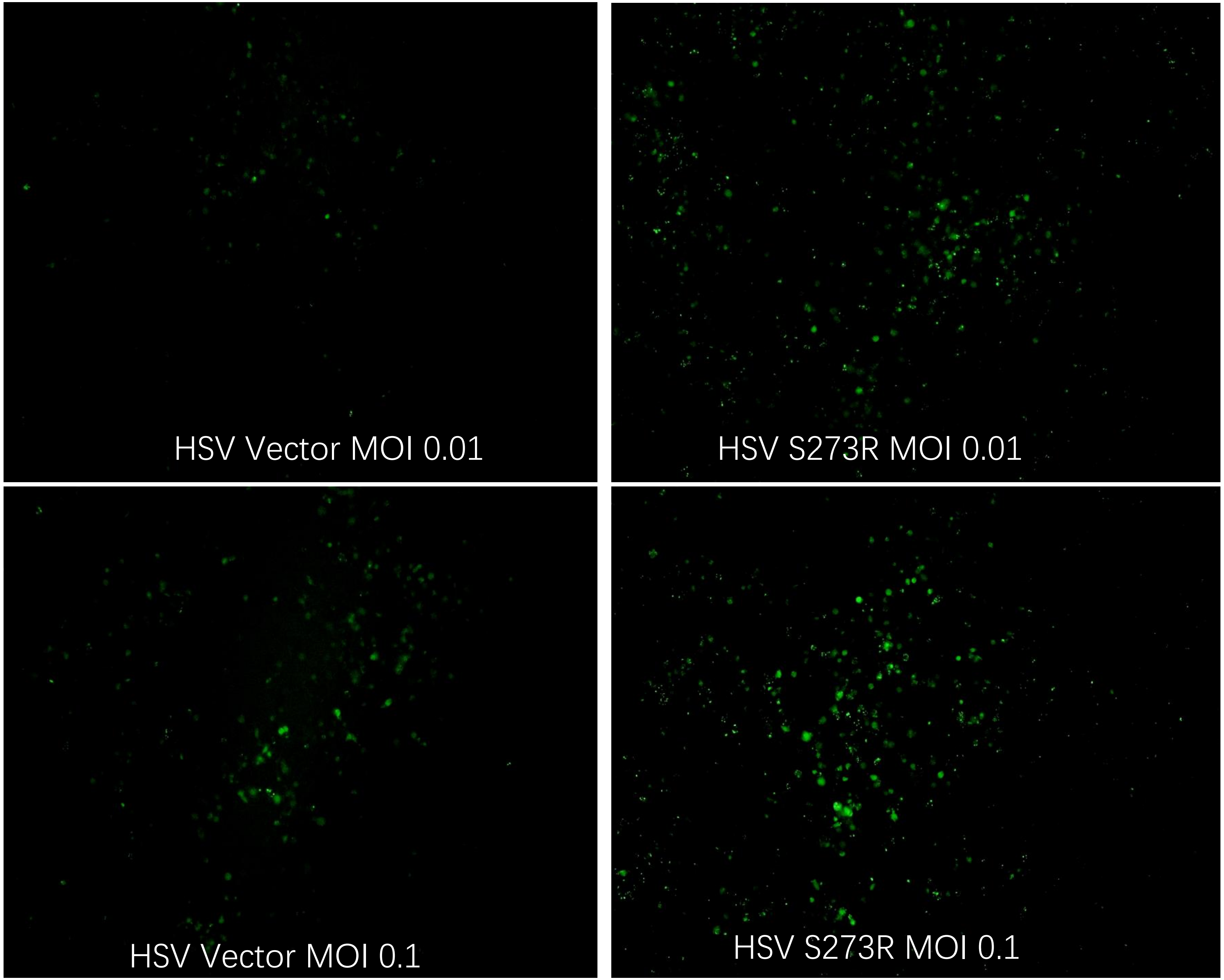

FIG4A

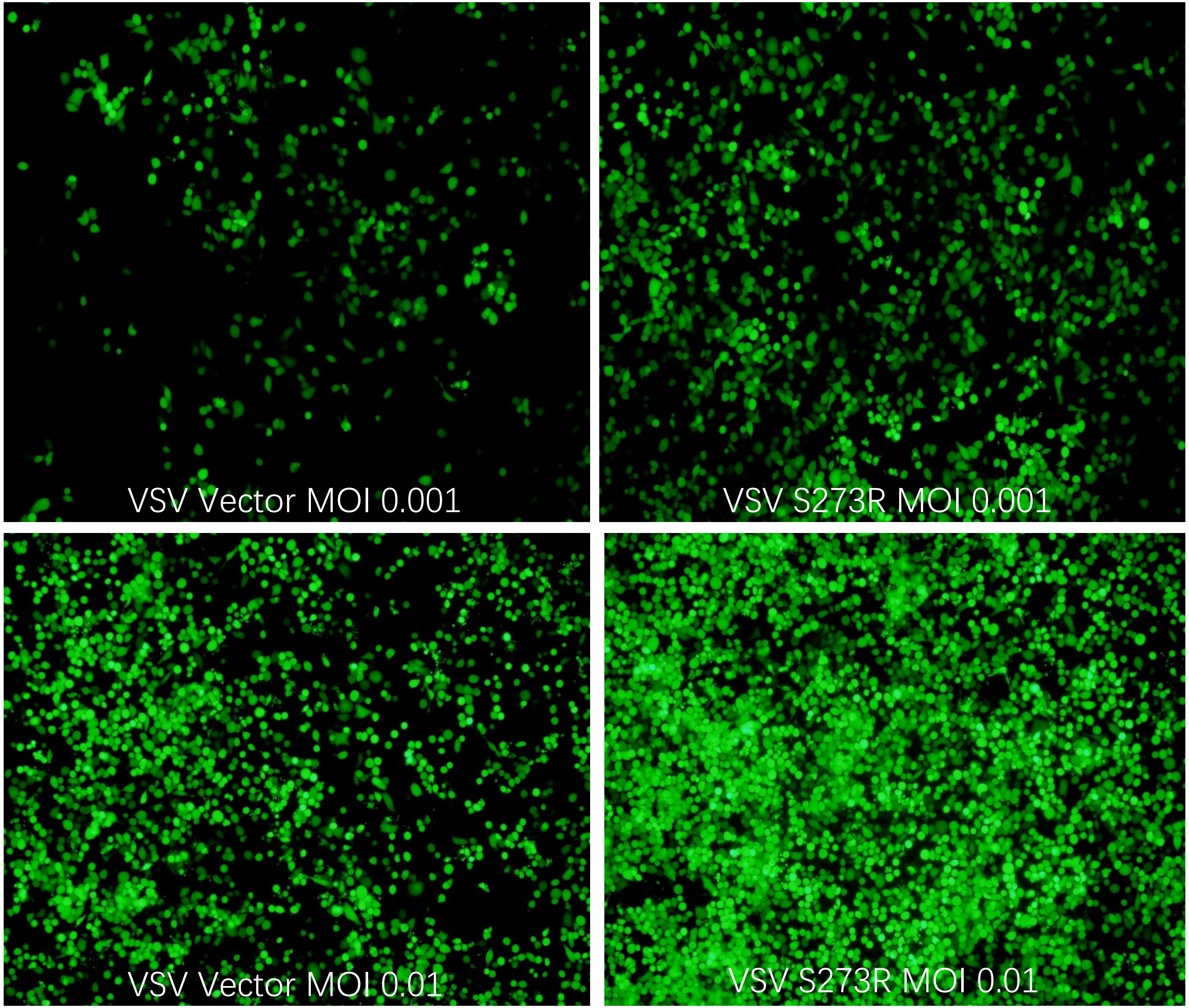

PAM cell

FIG5A

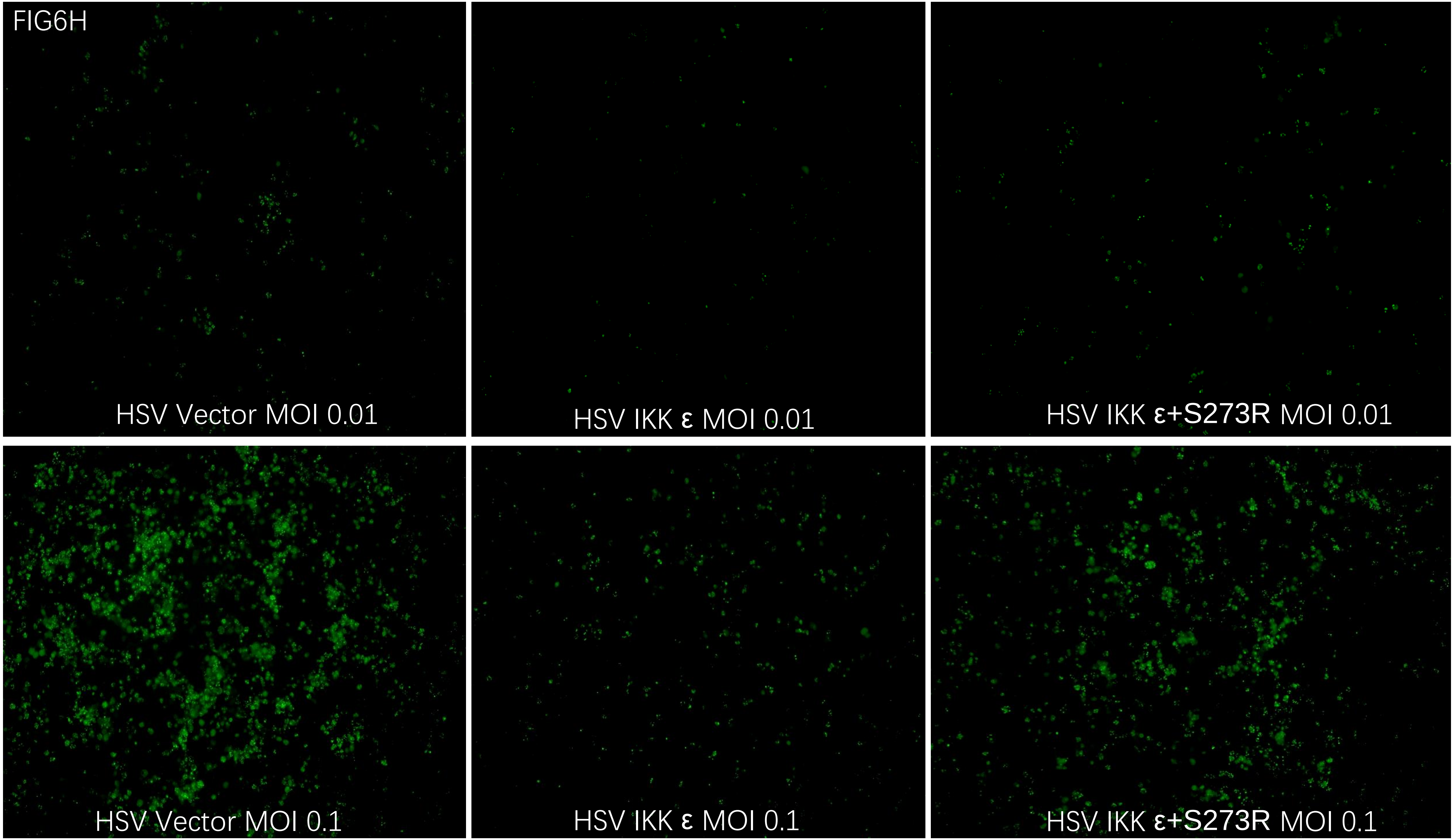
FIG6H
HSV Vector MOI 0.01
HSV IKK ε MOI 0.01
HSV IKK ε+S273R MOI 0.01
HSV Vector MOI 0.1
HSV IKK ε MOI 0.1
HSV IKK ε+S273R MOI 0.1

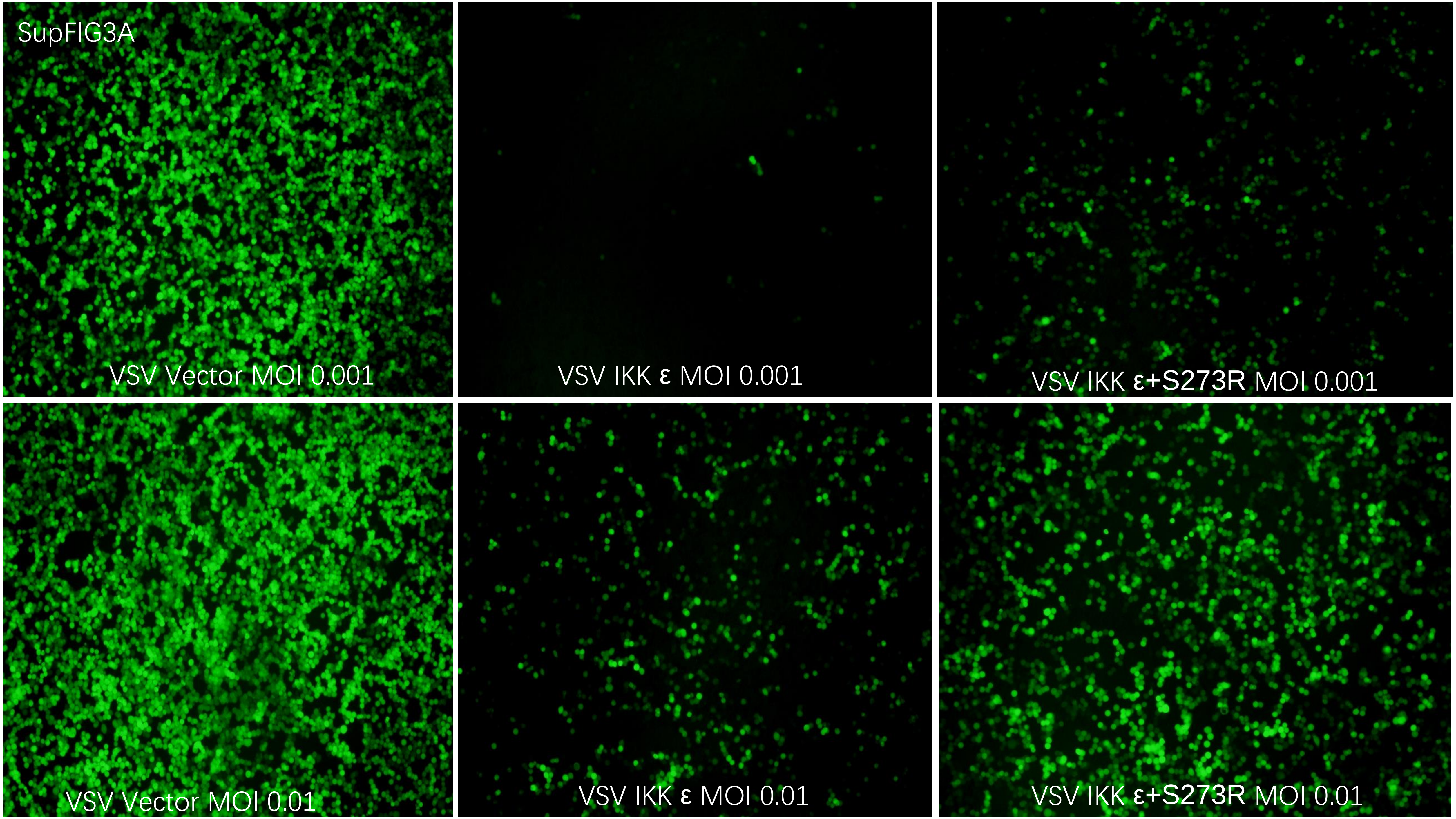
SupFIG3A
VSV Vector MOI 0.001
VSV IKK ε MOI 0.001
VSV IKK ε+S273R MOI 0.001
VSV Vector MOI 0.01
VSV IKK ε MOI 0.01
VSV IKK ε+S273R MOI 0.01
