# Supplementary material for: The African swine fever virus protease pS273R inhibits DNA sensing cGAS-STING pathway by targeting IKKε
Source: Virulence. 2022 May 1;13(1):740–56. doi: 10.1080/21505594.2022.2065962 (PMC9067533; doi:10.1080/21505594.2022.2065962)
Supplement: Supplemental Material [file KVIR_A_2065962_SM4607.zip › supplementary/Supplemental Material_Clean copy.docx]

**
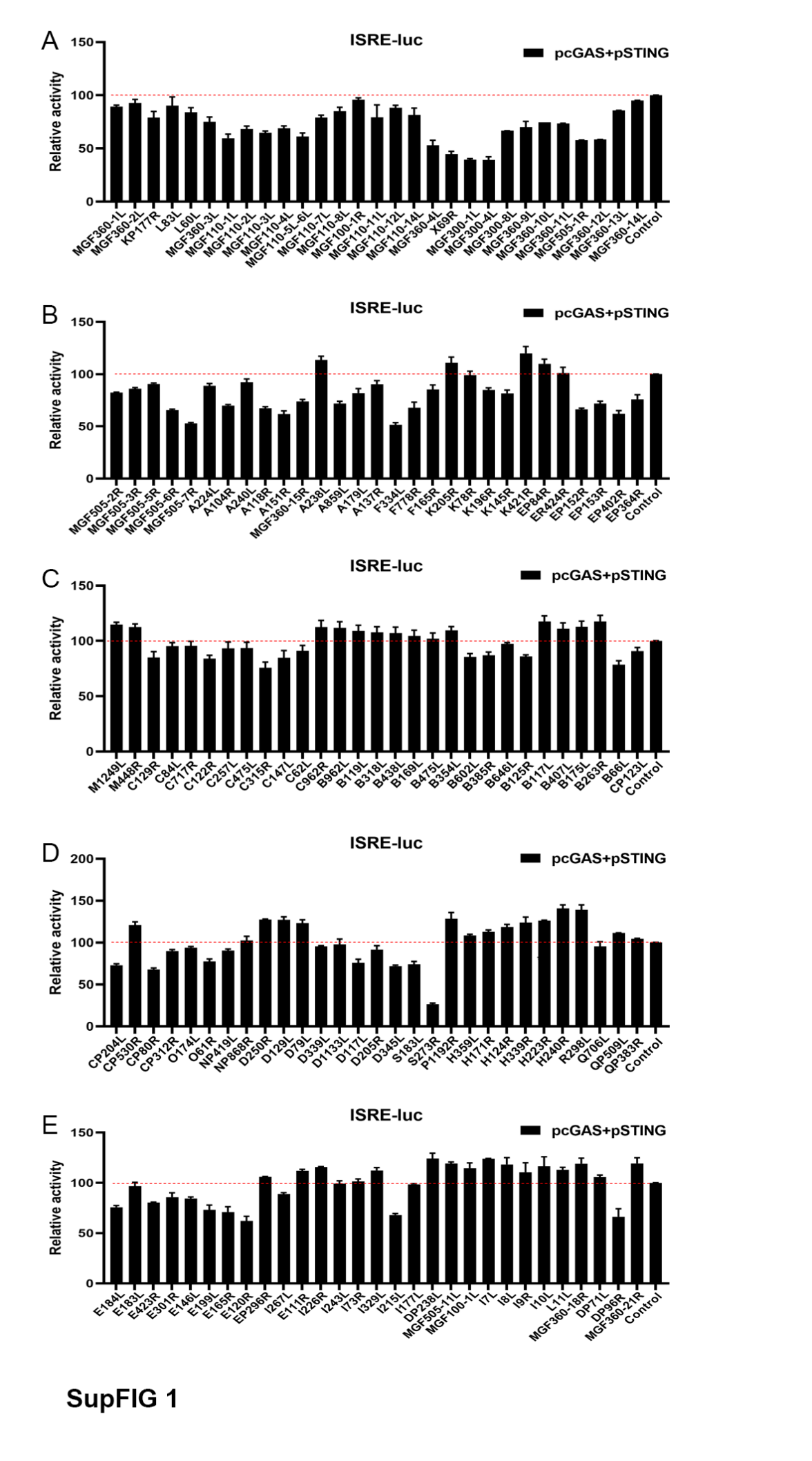
**

**Supplementary Figure 1. The screening of ASFV genomic ORFs for the modulation of porcine cGAS-STING signaling by ISRE promoter assay in transfected cells.** (A) HEK293T cells in 96-well plates (2×10^4^ cells/well) were co-transfected with 20 ng cGAS-HA and 10 ng STING-GFP, plus 10 ng ISRE promoter plasmid and 0.2 ng pRL-TK plasmid, along with 10 ng each ASFV ORF genes or empty vector 3×FLAG-pCMV, which were normalized to 50 ng/well by vector 3×FLAG-pCMV. At 24h post-transfection, luciferase activities were detected using Double-Luciferase Reporter Assay.


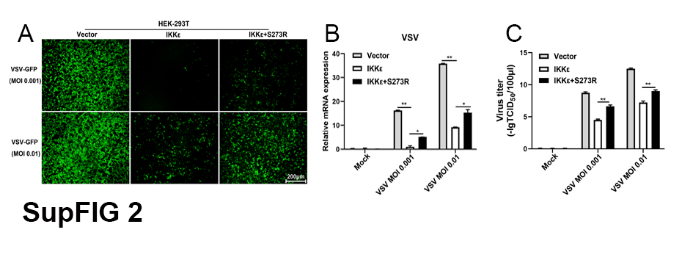


**Supplementary Figure 2.** **pS273R interfered the anti-VSV activity by IKKε.**

The IKKε (500 ng) were co-transfected with 500 ng pS273R plasmid or control vector into 293T cells for 24h, then the cells were infected with 0.001 MOI or 0.01 MOI VSV for 16 h, the GFP signals were observed by fluorescence microscopy (A). The infected cells were harvested to measure the VSV gene expression by RT-qPCR (B). The viral titer in the supernatant from VSV infected 293T cells was measured by TCID50 assay (C).


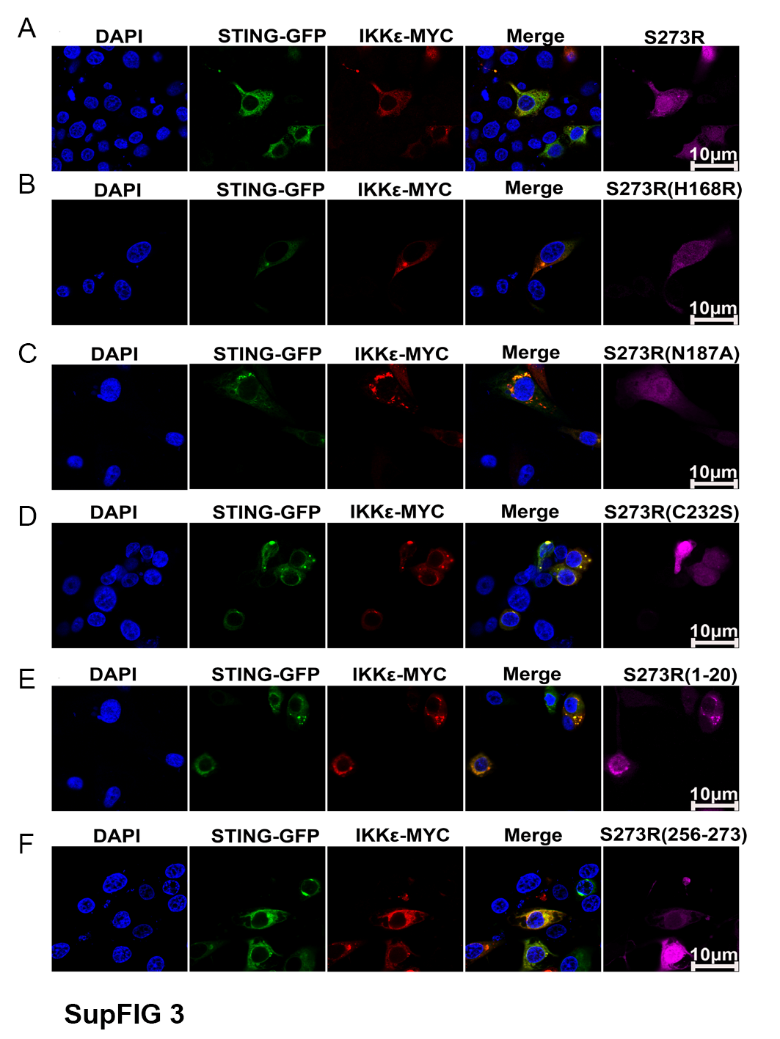


**Supplementary Figure 3. The cellular co-localizations between STING and IKKε in the presence of S273R and its various mutants.** 0.4 μg of each pS273R mutants or pS273R were co-transfected with STING-GFP (0.4μg) and IKKε-MYC (0.4μg) into PAMs for 24h, and then the cells were fixed, stained and examined for cellular co-localization by con-focal microscopy.





**Supplementary Figure 4.** **The inhibition of cGAS-STING signaling mediated downstream gene inductions by 2-D08.** (A-B) PAMs in 24-well plates (3×10^5^ cells/well) were stimulated with polydA:dT (1μg/ml) (A) or 2’3’-cGAMP (2μg/ml) (B) for 8h and then treated with 2-D08 (50μM, 100μM) for 18h. (C) HEK293T cells in 24-well plates (3×10^5^ cells/well) were co-transfected with cGAS-HA (0.5μg) and STING-GFP (0.5μg) for 24h, and then treated with 2-D08 (50μM, 100μM) for another 18h. The cells were harvested and subjected for analysis by RT-qPCR.


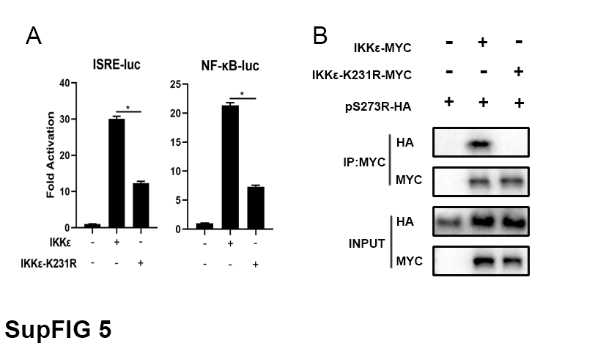


**Supplementary Figure 5.** **IKKε K231R mutant loses the signaling activity and the interaction with pS273R.** (A) HEK293T cells in 96-well plates (2×10^4^ cells/well) were co-transfected with 20 ng IKKε and 20 ng mutant K231R, plus 10 ng ISRE-luc or NF-κΒ-luc and 0.2 ng pRL-TK plasmid, which were normalized to 50 ng/well by control vector. At 24h post-transfection, luciferase activities were measured. (B) The HEK293T cells in 6-well plates were transfected with 1μg S273R-HA plasmid and 1μg IKKε-MYC or IKKε K231R-MYC for 48h and then cells were harvested and immunoprecipitated with anti-MYC antibody and both immunoprecipate and input samples were subjected to Western blot analysis using the indicated antibodies.

**Supplementary Table 1: The PCR primers used for gene cloning and mutations**

| **Primer names** | **Primer sequences** |
| --- | --- |
| **pCAGGS-S273R** | F: CTGTCTCATCATTTTGGCAAAGAATTCATGTCTATATTAGAA |
|  | R:ATCGTATGGGTAGCTGGTGATATCTGCGATGCGAAACAGAG |
| **H168R** | F: AGATGGCTACCCAGCGTTTTCCAGTGCCCG |
|  | R:CGGGCACTGGAAAACGCTGGGTAGCCATCT |
| **N187A** | F:AATTTCCCGTCGAAGCAAAATATTCGATGCTCCAGCAGTCGC |
|  | R:GCGACTGCTGGAGCATCGAATATTTTGCTTCGACGGGAAATT |
| **C232S** | F:GTAGGGGCCGCTCTCGGTCTGCG |
|  | R:CGCAGACCGAGAGCGGCCCCTAC |
| **∆N1-20** | F: CTGTCTCATCATTTTGGCAAAGAATTCATGAAAGATAGCTGT |
|  | R: ATCGTATGGGTAGCTGGTGATATCTGCGATGCGAAACAGAG |
| **∆N256-273** | F: CTGTCTCATCATTTTGGCAAAGAATTCATGTCTATATTAGAA |
|  | R:AACATCGTATGGGTAGCTGGTGATATCAGCGGATATAAAATG |
| **IKKε K231R** | F:TACATGATCTCCCTGTTCCGCCGTGGCCC  R:GGGCCACGGCGGAACAGGGAGATCATGTA |

**Supplementary Table 2: Primers for RT-PCR and RT-qPCR in this study**

| **Primer names** | **Primer sequences (5’-3’)** |
| --- | --- |
| **hIFN-β** | F: TGGGAGGATTCTGCATTACC |
|  | R: CAGCATCTGCTGGTTGAAGA |
| **hISG56** | F: CGCTATAGAATGGAGTGTCCA |
|  | R: TTTCCTCCACACTTCAGCA |
| **hISG60** | F: AGTCTAGTCACTTGGGGAAAC |
|  | R: ATAAATCTGAGCATCTGAGAGTC |
| **hIL-8** | F: GTTTTTGAAGAGGGCTGAGAATTC |
|  | R: CATGAAGTGTTGAAGTAGATTTGCTTG |
| **hRPL32** | F: CAACATTGGTTATGGAAGCAACA |
|  | R: TGACGTTGTGGACCAGGAACT |
| **pIFN-β** | F: TGAGCATTCTGCAGTACCTGA |
|  | R: CCGGAGGTAATCTGTAAGTCTGT |
| **pISG56** | F: ATGGGAGTTGGTCATTCAAGA |
|  | R:CAGGTGTTTCACATAGGCCA |
| **pIL-8** | F: CTGCAGTTCTGGCAAGAGTAAGT |
|  | R: CACTCTCAATCACTCTCAGTTCCT |
| **pβ-actin** | F: ATGAAGATCAAGATCATCGCG |
|  | R:TCGTACTCCTGCTTGCTGATC |
| **HSV1 gB** | F: TTCTGCAGCTCGCACCAC |
|  | R: GGAGCGCATCAAGACCACC |
| **VSV Glycoprotein** | F: GAGGAGTCACCTGGACAATCACT |
|  | R: TGCAAGGAAAGCATTGAACAA |
| **ASFV p72/B646L** | F: TCTCTTGCTCTGGATACGTTAATATGAC |
|  | R: CGGGTGCGATGATGATTACC |
| **ASFV S273R** | F: TTCTCCTCCAGGTCCCGTTATTCGTTGGATGGA |
|  | R: CACGTTGTCGAGGCGTGCCCTGATGTAAAACAG |

**Note: h denotes human; p denotes porcine.**

**Supplementary Table 3: ASFV S273R siRNA sequences in this study**

| **Primer names** | **siRNA sequences (5’-3’)** |
| --- | --- |
| **S273R siRNA 1** | A: GGUAUAUAAGGGAGAAGAGTT |
|  | AS: CUCUUCUCCCUUAUAUACCTT |
| **S273R siRNA 2** | A: GCGCAGAGCAUCUUACAAATT |
|  | AS: UUUGUAAGAUGCUCUGCGCTT |
| **Control siRNA** | S: UUCUCCGAACGUGUCACGUTT |
|  | AS: ACGUGACACGUUCGGAGAATT |
