# Supplementary figures and images for: The African swine fever virus protease pS273R inhibits DNA sensing cGAS-STING pathway by targeting IKKε
Source: Virulence. 2022 May 1;13(1):740–56. doi: 10.1080/21505594.2022.2065962 (PMC9067533; doi:10.1080/21505594.2022.2065962)

FIG9A

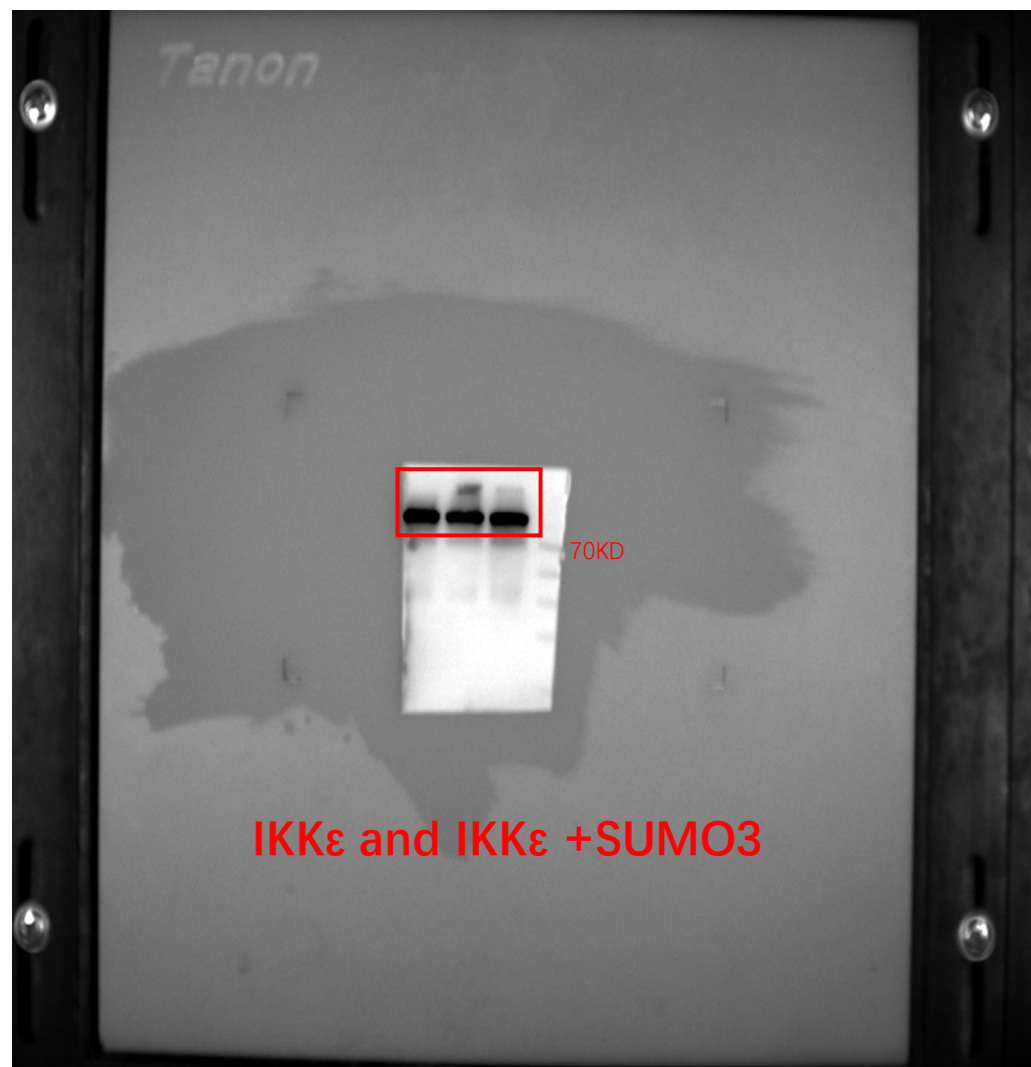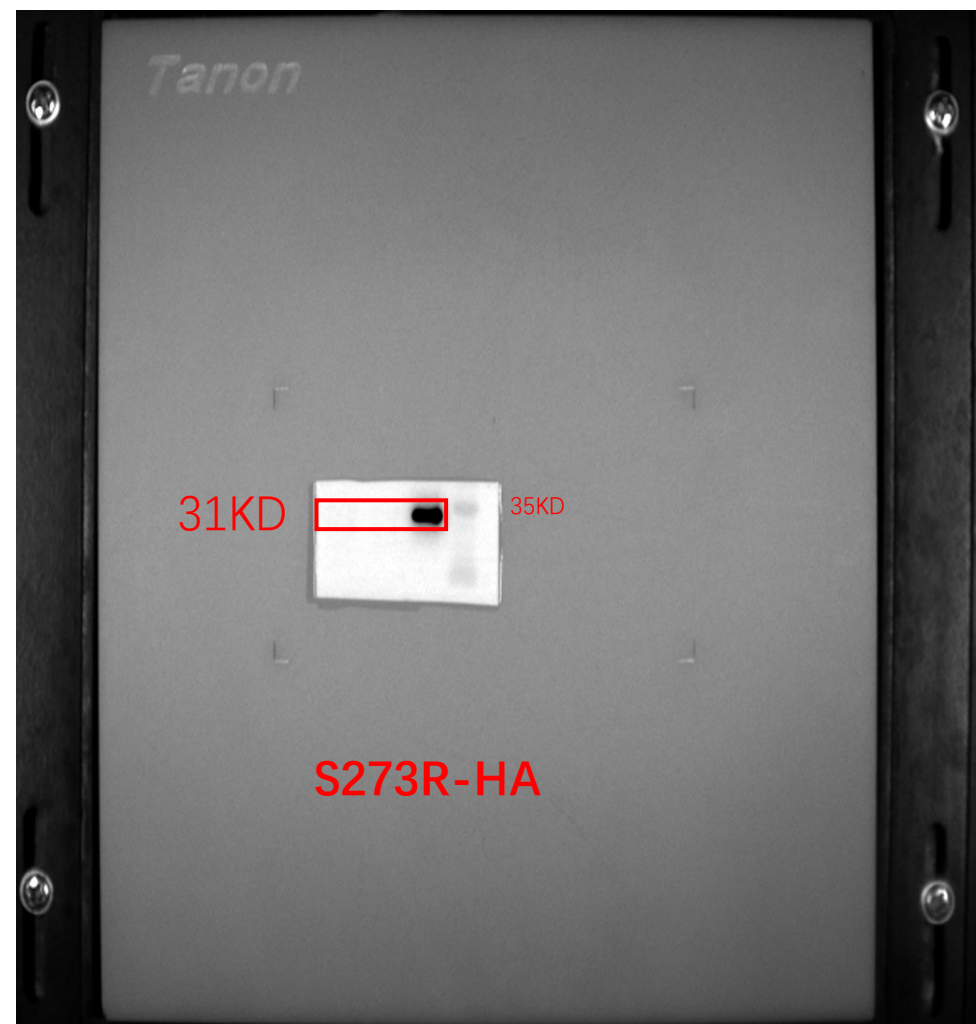

FIG9A

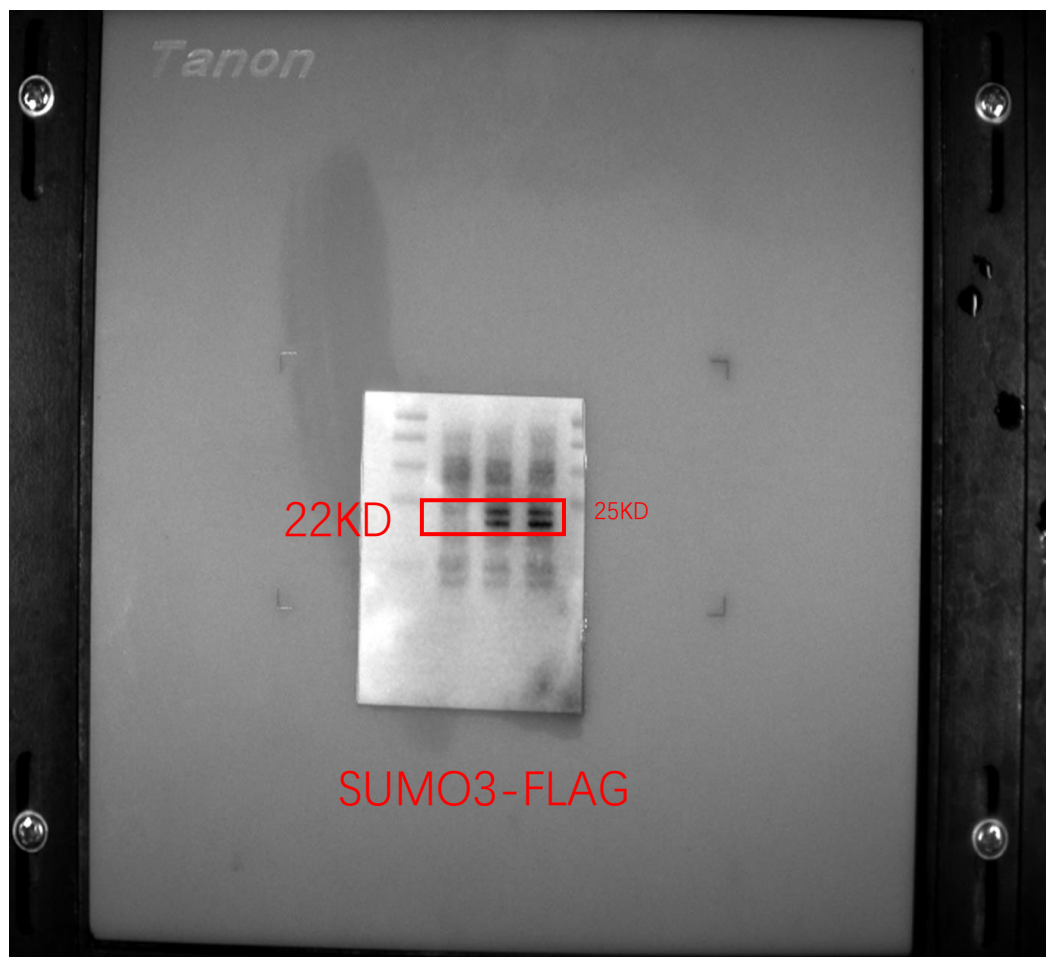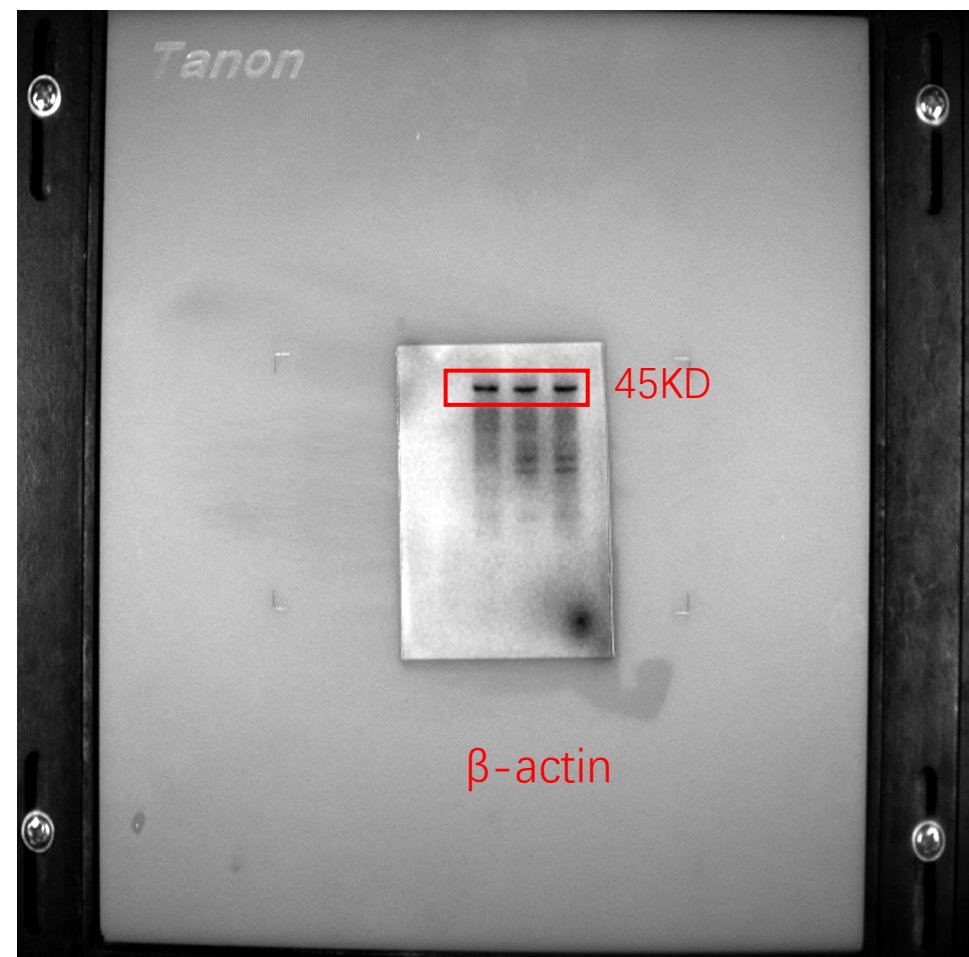

FIG9G

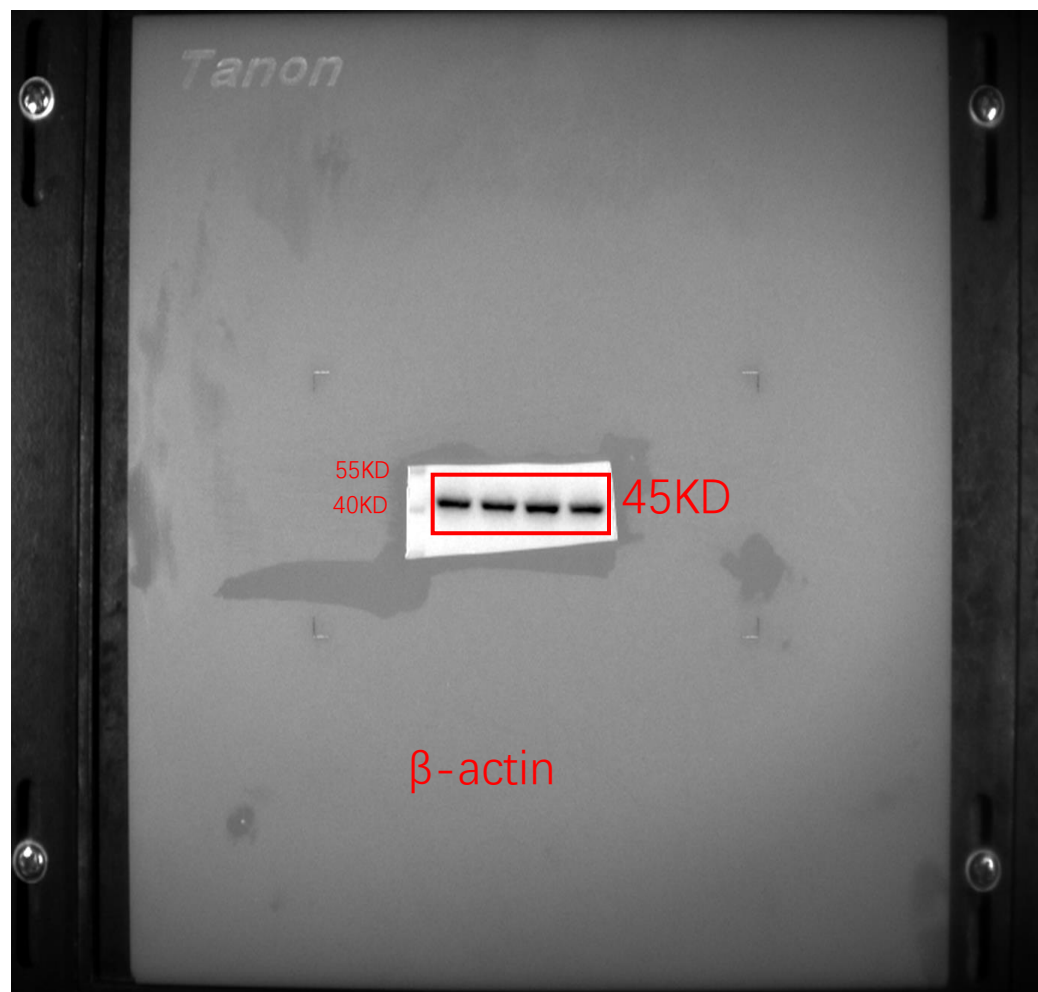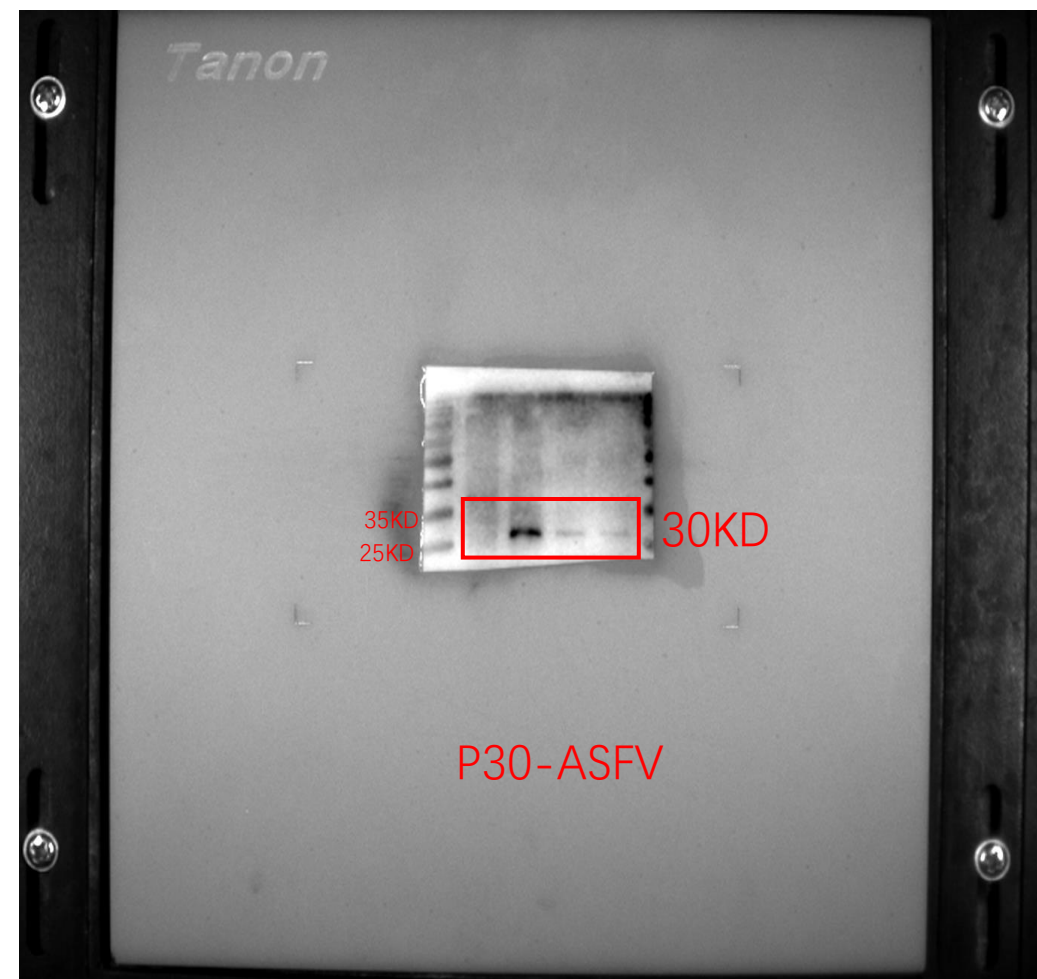

Supplement: Supplemental Material [file KVIR_A_2065962_SM4607.zip › supplementary/New raw WB data.pdf]

FIG9D

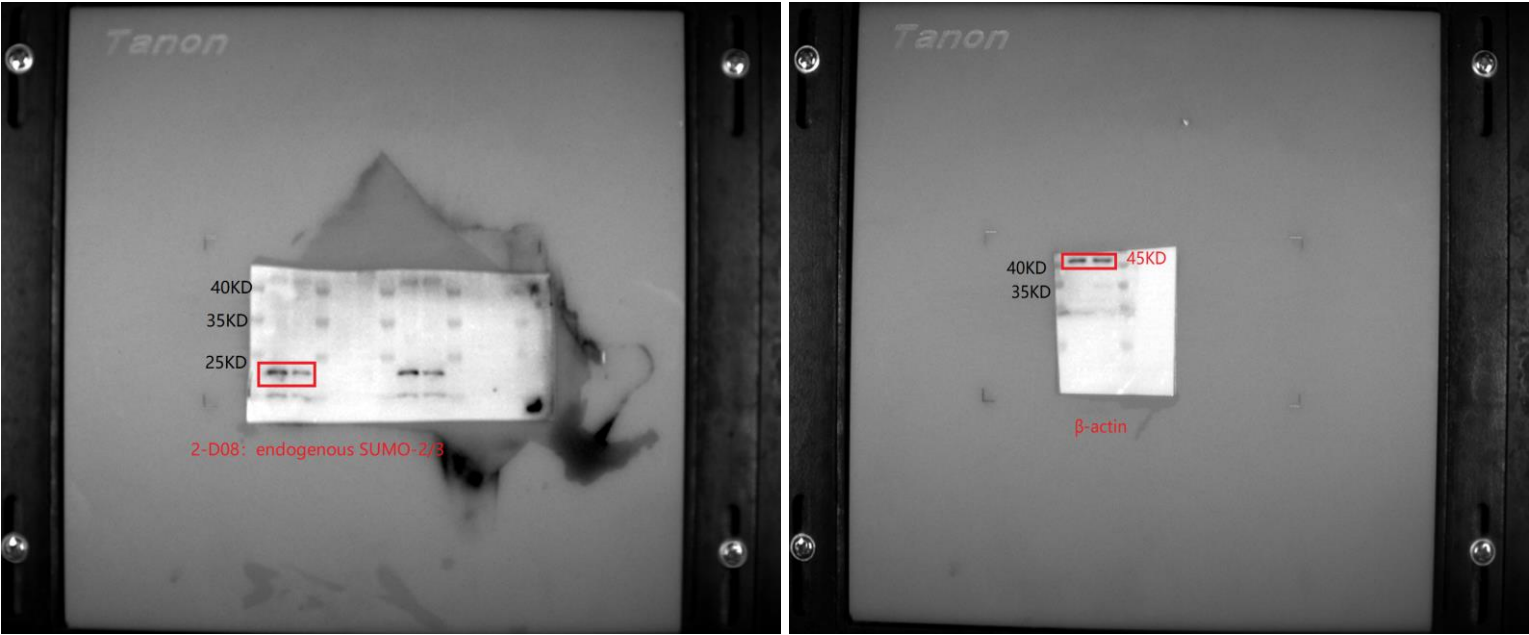

SupFIG5

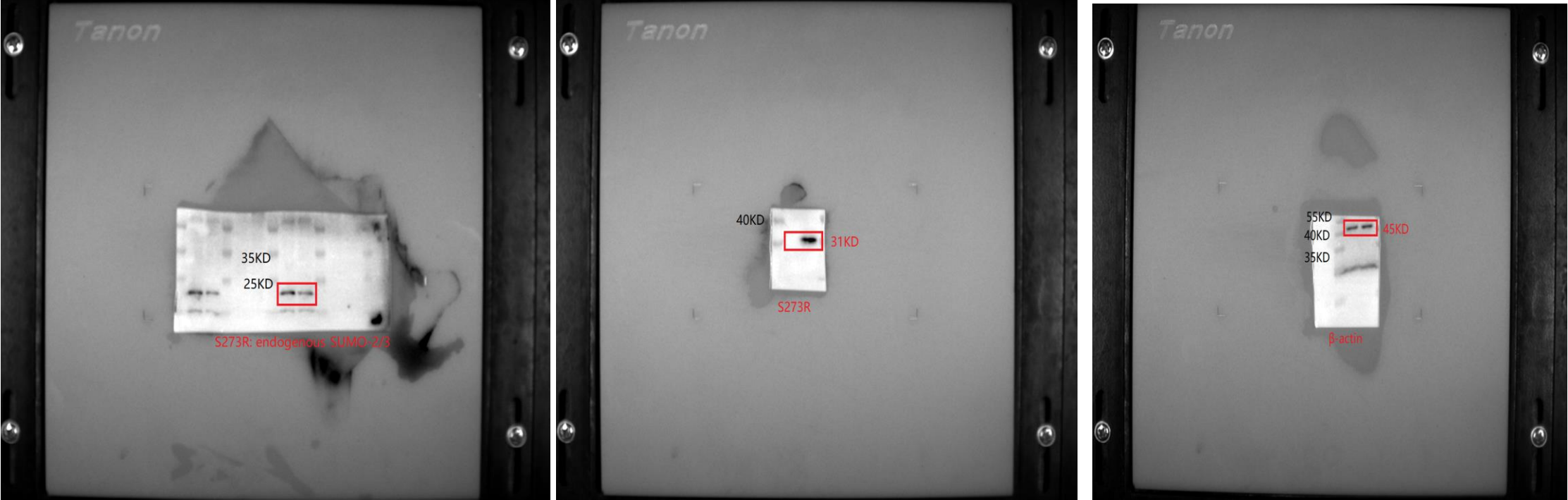

SupFIG6

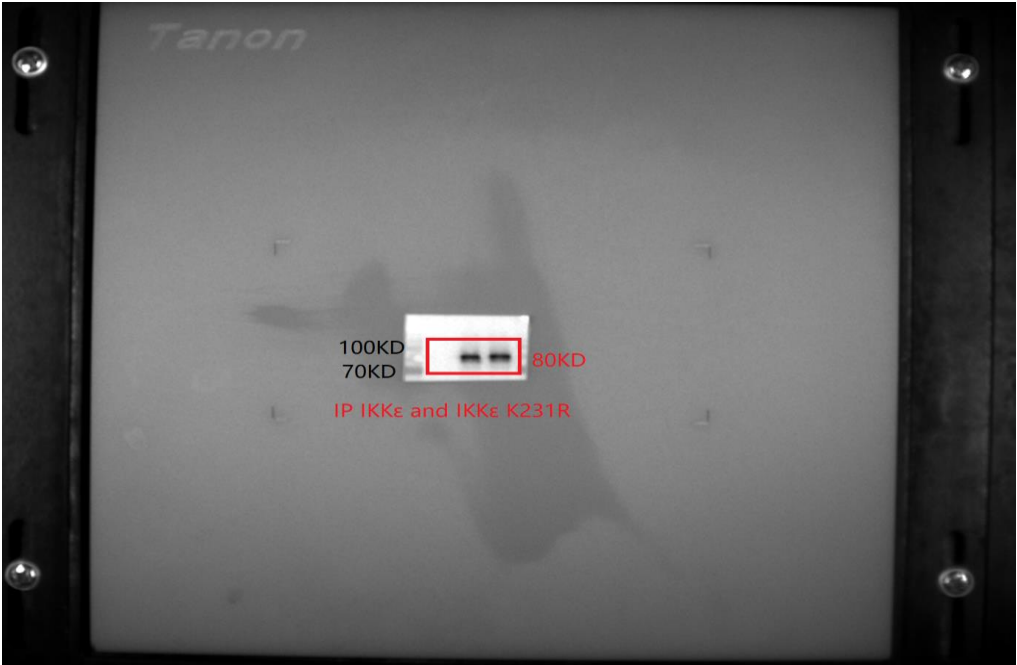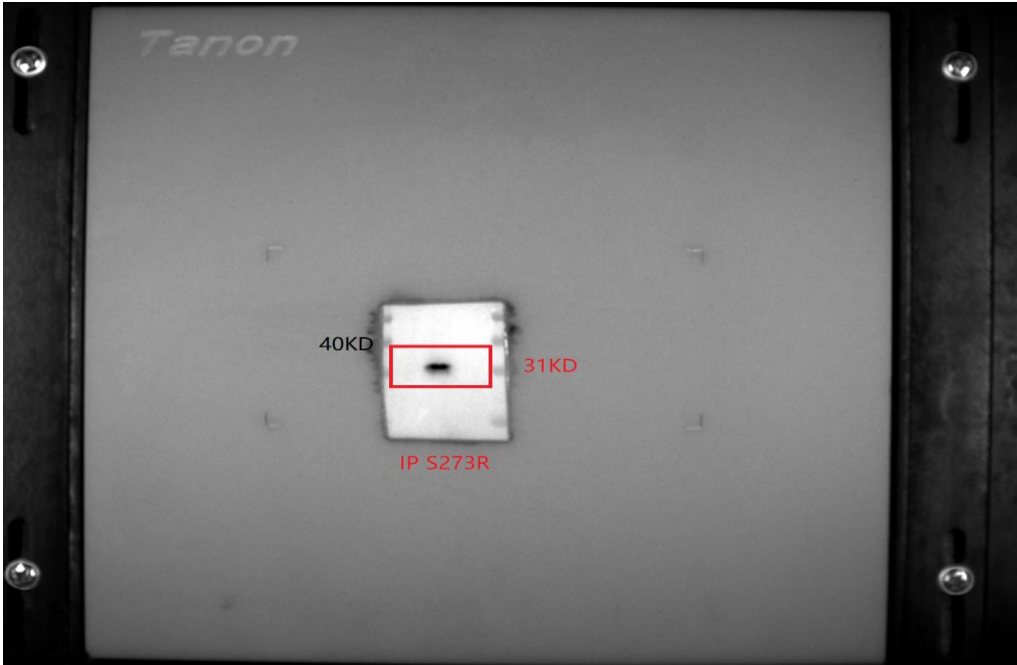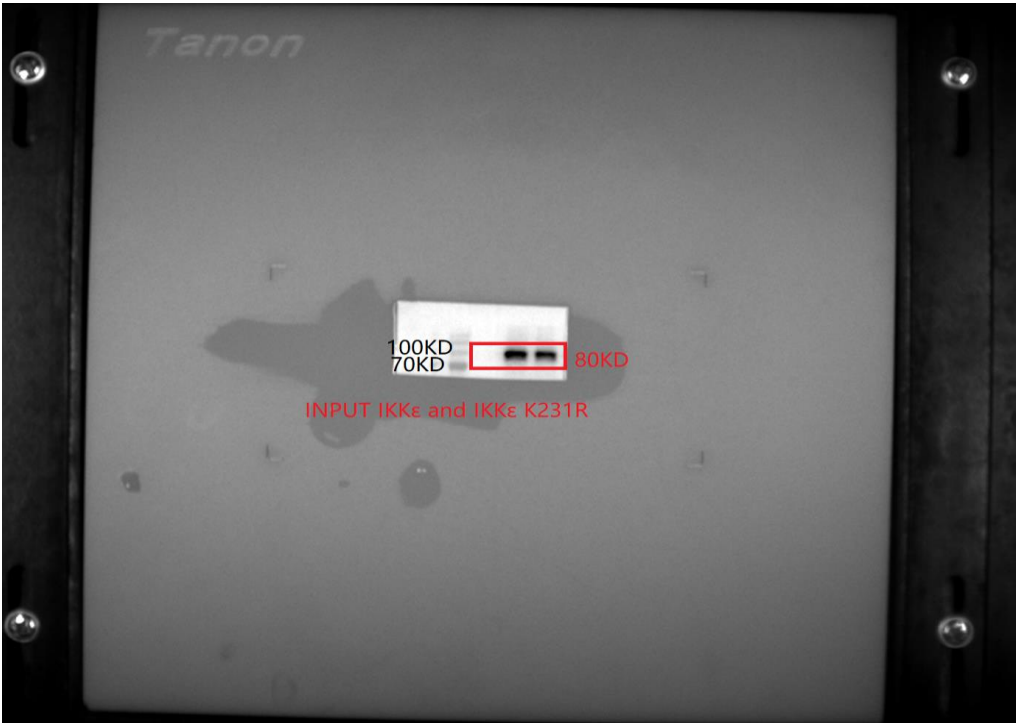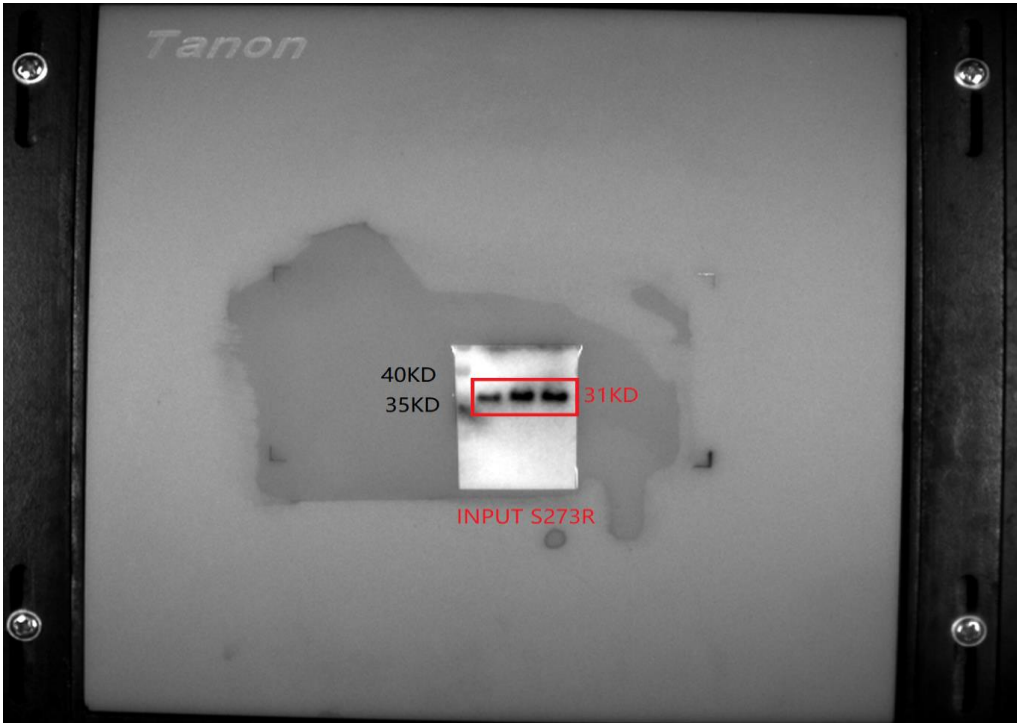

Supplement: Supplemental Material [file KVIR_A_2065962_SM4607.zip › supplementary/Raw data of WB Fig 9D, SupFig 5 and 6.pdf]
